# The effects of narrative framing of own broken love on understanding the past and imagining the future in close relationships

**DOI:** 10.1371/journal.pone.0334973

**Published:** 2025-11-25

**Authors:** Jolanta Zuzanna Czarnecka, Jerzy Trzebiński

**Affiliations:** Department of Psychology, SWPS University, Warsaw, Mazowsze, Poland; Public Library of Science, UNITED KINGDOM OF GREAT BRITAIN AND NORTHERN IRELAND

## Abstract

The project aimed to assess the effects of narrative framing applied to one’s past romantic relationship. It was expected that the activation of self-story on broken love would lead to more beneficial thinking about the personal past and possible future in close relationships. Women after the romantic relationship breakup (N = 422, 18–30 years old) took part in a two-stage naturalistic experiment in which they were randomly assigned to two sets. The narrative set participants wrote a story of broken love, while the control set participants answered questions about the past relationship. After seven days, participants declared their current thoughts and breakup understanding and provided open responses on the breakup reasons and thoughts about the future in close relationships. Self-story activation led to higher reflection elaboration and greater focus on causal connections, actions, and appropriate time perspectives. Women with activated self-stories provided more coherent descriptions of breakup reasons, declared a higher understanding of breakup, and experienced more future-oriented thoughts. The presence of the reported effects differed with the mode of exploration of the self-story activation level, and most of them were positively related to the level of narrative organization (plot structuring) of the narrative set participants’ self-stories. These results underscore the impact of narrative thinking on cognition, and suggest that self-story writing interventions could help manage challenging interpersonal experiences.

## Introduction

### Narrative thinking on broken love

When something difficult happens, people tend to put in into words and share it with others [1–4]. It is mostly done by presenting a story: who did what, what for, what obstacles occurred, how they were tackled, and how it all ended [5,6]. People resort to the stories not only for communication and social organization [7,8] but also to find meaning in their experiences [5,9–12]. The overbearing presence of narrative in human thinking remains unmatched with our understanding of its influence on functioning and perception [9,13–15]. Stories we hear and share are not objective reports of events [16,17]. They are intricate cognitive constructions. Narrative thinking starts with the acquisition and presence of narrative cognitive schemata. Thanks to these, personal experiences can be formed into a self-story through narrative framing. After gaining such an organization, the story might be present in one’s mind (inner narrative), shared (told narrative), and exert regulatory power on functioning [9,18,19]. The moment the participant is asked to share or think of a self-story is called the activation of the self-story. This term highlights the role of this activity in general participants’ narrative thinking, the level of which is intensified when attention is paid to the construed narrative.

The activation of a self-story has been shown to improve various areas of psychological functioning. This may lead to anxiety reduction, increased stress-related growth and meaning in life for widowed participants [20]. For mothers raising children with autism, creating a motherhood self-story can increase the meaning of life, self-esteem, and a wide range of well-being measures (ranging from positive affect and optimism to hope) [21]. Experimental disclosure studies have predominantly focused on trauma and health-related issues [22]. Consequently, there is a significant gap in our understanding of how narrative framing affects more common challenging experiences, such as romantic relationship breakups. In the present study, we verified the effects of narrative thinking by observing the consequences of the narrative framing of broken love on the after-breakup cognition of emerging adult women. Despite the evident necessity for interventions tailored to the needs of individuals experiencing romantic relationship breakup [23–26], targeted scientific investigation of such interventions is scarce [25,27,28]. To the best of our knowledge, no studies have experimentally verified the time delayed effects of self-story creation of a romantic relationship breakup. However, the beneficial effects of narrative framing have been demonstrated in the case of divorce [29,30]. Participants who created the story of their divorce compared to the control and expressive writing groups experienced beneficial changes in heart activity: decreased average heart rate and increased heart rate variability in measurement after averaged time of 7.5 months [30]. In the area of psychological functioning, the first analysis showed that for participants experiencing high rumination levels and actively looking for meaning, self-story activation and expressive writing had an iatrogenic effect compared with the active control group task [31]. However, in-depth examinations revealed that gaining a narrative understanding might be helpful for post-divorce emotional recovery. The results showed that a higher declarative finding of the narrative frame, through which separation could be understood, predicted lower psychological distress. Next, mediation between psychological overinvolvement through narrative understanding (called in a source a narrative coherence) and psychological distress differed among conditions. In the self-story activation group, when narrative understanding was considered, no negative relationship was found between psychological overinvolvement and distress. Simultaneously, it remained significant under the expressive writing condition. This led the authors to state that the first reported negative influence of self-disclosure [31] should be attributed mainly to expressive writing activity [29].

### Tracking the effects of the self-story activation

Previous research on self-story activation has explored the broad effects of narrative thinking, such as alterations in life’s meaning or physical health [20,30]. In our experiment, we tried to more directly observe the processes involved in the positive impact of narrative framing of difficult life situation.

The primary function of self-story creation is to generate meaning to facilitate an individual’s understanding of their experiences [5,9–12]. Achieving such a goal lies in processes initialized with narrative framing of experiences and enthroned by it, operating in mutual interaction, and driving each other forward. Experiences are ordered by fitting them into a canonical narrative structure [5,32]. At the same time, they have meaning asserted, which comes from both the role in the story and the connection of events subordinated to the goal of constructing a coherent plot structure. The low-level process present here establishes causal connections between events [19,33,34], not necessarily real, but based on life similarity and participants’ tendency toward narrative representation of events [5,9]. We suspect that this effect is transferred to the ability to construct coherent reflections connected with the narrated content. The second anticipated outcome stemming from narrative framing is an increased subjective feeling of understanding areas connected with the scope of the self-story, as meaning is felt when the stimuli are ordered and coherent [35].

The cognitive processes elicited by narrative thinking pertain to the personal past and the imagined future. This notion is postulated in narrative theory [9,18] and can be connected to the mechanism of mental simulation [36]. The act of “imagination and the generation of alternative realities” [37, p. vii] lies heavily on episodic memories that are seen as building blocks for past recollections and future prospects [38,39]. Narrative thinking refers to concrete episodes retrieved from episodic memory and reassembled [19,40] for understanding purposes, including uncovering the core of processes revealed over time [13,41].

In thinking effectively in the area of the past, narrative thinking should result in improved retrieval of events important to the reflection topic, visible by higher elaboration stemming from the higher motivation to explain events through cause-effect connections [34] and the increased availability of episodic memories. A study on narrative mindset effectiveness in problem solving showed that participants with induced narrative thinking without being asked to do so included more personal experiences (episodic memories, thoughts, beliefs) in the given pieces of advice on how to solve problems, and that their presence supported the effectiveness of the suggested solution through empathy for the recipient [42]. Costabile and Klein [34] demonstrated that narrative thinking improves inference generation in past explanations and future predictions. Participants were presented with a list of two actors’ unrelated actions and were given different processing instructions. Among these, narrative instruction (creating a narrative from presented sentences) emerged as the best or one of best in evoking elaboration on material (general inferences) and construing causal connections (narrative inferences). Next, the researchers examined forward (future) directed inferences. To guarantee their spontaneity and non-forced nature, they used non-direct measures of words activated in the participants’ thoughts. Compared with the memorization condition, the narrative group participants showed a higher presence of predicted content in their minds, with no difference in control measurements. This leads to the probable conclusion that, in real-life settings, activated narrative thinking will evoke numerous future-oriented thoughts that should be closely related to the self-story content, which in the situation of a romantic relationship breakup would be future in close relationships.

Three observation methods were selected based on their distinctive advantages in achieving the study’s objective of tracking changes in post-breakup cognition after self-story activation: self-reports, trained coder assessments, and language-use analyses. Self-report measures can be more predictive of functioning than scores assigned by trained coders [43] while being at risk of a lack of connection with participants’ psychological reality [44]. Trained coders assessments can encompass various psychological processes in a given content [42,45] and provide insights into narrative structural properties [20,46]. Finally, language use is a sign of activated cognitive processes [47] and has been proven to be susceptible to narrative mindset activation [42]. We will examine reflection length (by use of the Linguistic Inquiry and Word Count: LIWC – Word Count category) [48,49] as an indirect measurement of elaboration and the use of words pointing to the creation of causal connections (LIWC – Causation). The next area will be the number of verbs (by use of the Literary Exploration Machine: LEM – Verbs category) [50] showing the action orientation of the reflection. Narratives use more concrete language than impressions of a person, including action depicting verbs [51]. Since narratives focus on actors’ intentions, this action orientation will likely to carry over to further reflections [36,52]. Finally, the context specificity of narrative thinking [14] may reemerge in better time orientation of reflection, seeing the past as past (reflection on breakup reasons – past tense verbs, LEM) and being free to explore the future (in future-oriented thoughts – future tense verbs, LEM) [50]. Costabile [51] showed that narratives (compared to impressions) referenced time and physical locations more frequently.

Examining the structural properties of shared self-disclosure is crucial when searching for mechanisms related to the intensity of previously described cognitive processes. The goal of creating a story from personal experiences is to facilitate their structurization into a narrative [29]. Canonical story structure definition, chosen by as at the base of capturing most of unique narrative characteristics is “The story features characters who have certain intentions and encounter obstacles to their realization. The events and actions happening in the story focus on overcoming the obstacles or influencing them, and the end of the story is success or failure in overcoming the obstacles.”([6, p. 36] self-translated; in similar tone: [5,53]). Non-uniformly defined narrative coherence is primarily a measure of how well a story is construed [46] or the level of narrative understanding gained in particular life areas [29]. Creating a coherent self-story has been widely proven to be positively connected with beneficial psychological functioning [54] and the higher likelihood of good social reception [1,55]. Among the various processes visible in an open account [45], we aimed to examine the level of embodying the canonical story structure, which we call plot structuring. Unable to find a precise tool to encompass the structure of long, multiepisodic narratives, we created a scale inspired by the structure subcomponent of the Baerger and McAdams [56] coherence scale and classical narrative theories [5,32,52]. An analogous approach in the analyses of Odachowska et al. [20] showed that plot structuring effects varied by its levels and time since self-story creation. The effects on anxiety were observed faster after the intervention (2 weeks), while on improvement of meaning in life only after three months. A low level of plot structuring is associated with a decrease in trait anxiety. In the medium-level plot structuring, state and trait anxiety decreased, and stress-related growth and purpose in life increased. In the highest level of plot structuring observed was a decrease in trait anxiety and increase in purpose in life. Higher levels of plot structuring have emerged as more beneficial. In other explorations, stories of mothers with younger children diagnosed with autism, who had less time to accommodate the family situation, had lower plot structuring, which shows that narrative thinking effectiveness may vary depending on the situation. Similarly, narrative structuring of self-stories was positively correlated with well-being measures of meaning in life, hope, self-esteem, optimism, positive affect, and stress-related growth [21]. On this basis, we suspect that the narrative plot’s structuring level will be positively connected with the observed narrative thinking effects.

The challenge in studies on self-story activation is the selection of the control condition. The natural inclination to think narratively results in submitting stories to nonnarrative tasks [57] and spontaneously creating narratives from loosely connected actions to help oneself predict future events [51]. When events are thematically, temporally, and causally linked, they are likely to be constructed as stories [58]. Events in a participant’s life meet these criteria, making their narrative representation likely independent of and preceding research intervention [5,18]. What can be observed in studies is self-story activation, where through tasks of thinking of, telling, or writing a self-story, we increase its regulative power on functioning by prompting its formulation into a coherent structure that subsequently attracts attention to the created narrative content for its consideration, reformulation, and improvement, and further use to formulate explanations of past, present, and future situations [11]. Various open tasks can facilitate self-story creation and the search for narrative meaning [31], necessitating a contrast in narrative thinking studies that actively reduces narrative thinking or minimizes self-story activation. In laboratory studies on narrative thinking, this goal is achieved by assigning control tasks, such as impression formation [36], memorization to halt mental elaboration [34], or creating categories and sorting stimuli by shared characteristics [42]. These tasks facilitate cognitive processing through generalization, abstraction, and decontextualization, as defined by Bruner [5] as the paradigmatic mode of thinking. This led us to choose the following modes for comparison: experimental/narrative, in which participants will write a self-story about their past relationship, and control, in which participants will answer open questions about it engaging in paradigmatic cognitive activities (listing, comparing, identifying). A similar approach has been used by Trzebiński et al. [21] and Odachowska et al. [20].

Another potential contrast that is yet to be thoroughly investigated is the intra-narrative group comparison between participants who presented a self-story and those who did not, despite being prompted to do so. Observing differences in self-story activation could highlight the effects of inability or unwillingness to engage in narrative thinking. The capacity to provide narrative accounts of significant life experiences correlates with positive psychological well-being [54] and is impaired in individuals with emotional disorders [59], borderline personality disorder [60], and brain malfunctions [61]. The precise mechanisms of narrative coherence disruption remain to be elucidated. Negative affect does not necessarily worsen narrative coherence [62] and the narrative coherence and emotional disorders are indirectly connected through ruminations [59]. Additionally, narrating difficulties may arise from situational characteristics in case of its stressfulness or ambiguity [21,63]. Therefore, examining cases of narrative creation failure is crucial for understanding the potential impact of difficulties in narrative thinking.

### Emerging adult women and romantic relationship breakups – contextual information on a group choice

Modern romantic relationships are characterized by instability [64–68]. Approximately 75% of individuals have experienced at least one breakup, with the majority encountering multiple breakups [69]. It is in the period of emerging adulthood (18–25/29 years of age) when individuals are expected to reach the developmental task of learning how to form long-lasting romantic relationships [70–72]. However, most importantly, emerging adults aim to establish their identity and position within the adult sphere [73,74]. The challenges of engaging in numerous personal life tasks, discovering oneself, and coordinating with a partner might lead them to postpone considering serious relationships [75] while experiencing rapid changes in their love life, transitioning between being single, engaging in short-term relationships, and forming committed bonds [68]. Breakups, which are often unavoidable, entail a diverse array of consequences. Their detrimental impact on functioning should never be underestimated, as it ranges from negative emotions [25,26], worse mental health [23] to suicidal tendencies [24]. However, ending a relationship is not universally bad, as a breakup from an unsatisfactory bond might benefit personal well-being [23,76]. Regardless of the nature of the breakup, when an individual engages in productive self-reflection regarding their role and the factors contributing to the relationship’s end, they may gain valuable insights from the experience [77] and potentially enhance their chances for maintaining fulfilling romantic relationships in the future [78].

The focus of this study was restricted to emerging adult women after the breakup from heterosexual, informal romantic relationships. This decision was informed by the objective of obtaining a homogeneous sample and the potential unique impact that romantic relationship termination may have on their life trajectories. Women more often initiate breakups [69]. Studies are inconclusive on which gender suffers more after them, but when significant differences are found, women are pointed out [23,69,79]. Women tend to care more to reflect on and find the meaning of difficult interpersonal events [80]. The qualitative study of Dalessandro and Wilkins [81] showed that women decide on changes in functioning in romantic relationships based on experiences from previous ones, while for men, the implemented behavioral adjustment tends to be a matter of subjective decision uninformed by prior events.

In particular cultures (current study conducted in Poland), genders might differ in their belief of family importance [82] and relationship attitudes, including reasons to stay in or break the unsatisfactory relationship [64,65,83]. Factors connected to unequal material situations [83] and relationship models burdening women with more responsibilities [84,85] further support the differentiation of female and male experience of the breakup. Since women generally enter marriages faster than men [74], they have less time to learn how to function in a romantic relationship, which can lead to higher importance of shaping experiences from the time of emerging adulthood.

It was hypothesized that narrative structuring would be advantageous for heterosexual women, as they have a cultural backdrop to understand a breakup and are not subject to potential homophobic or indifferent reactions from a heteronormative society while sharing breakup self-stories ([86], for polish context: [87]). These two factors may be associated with the potential effects of activating a self-story, which resulted in deferring the exploration of non-heterosexual relationships for future investigations.

In the current analysis, only breakups from informal relationships were analyzed, and participants were required to be maidens (never married). Divorces have been a greater focus of scientific exploration than informal romantic breakups [27]. These two relationship types differ significantly in factors counteracting their breakup (such as legal difficulties in obtaining a divorce, the belief – significant for part of the Polish population – that a marriage cannot be broken up, which may stabilize some unhappy marriages [65,67]). Those with less to lose from of the relationship breakup (being in an informal relationship or being single) tend to be more open to it [65,67,83]. Since informal relationships are gaining increasing importance at the personal and societal level (for Polish context: [88,89]), analyses of personal functioning connected with them is much needed. They are also more important category for emerging adults, as a possible bridge to the marriage [64]. The condition of being a maiden was based on the assumption in the experience of the divorce would affect how one would view breakups from the next, informal relationships. Polish divorced individuals are the highest supporters of the right to divorce from all marital status groups, with level similar to never married individuals [67].

The final requirement of staying single after breakup was based on the assumption that entering a new bond might change how one sees the previous relationship, which would further differentiate the consequences of its narrative structuring. Changes in the perceptions of broken love are indirectly supported by differences in thematic contents of breakup self-stories [90] and the higher well-being of those in a new bond [91].

In Polish culture, close relationships are highly valued, which makes it an ideal population for studying the effects of narrative framing of broken love. Family happiness is one of the most important everyday life values, receiving the most indications (being pointed out by 80% of respondents) [82]. This is accompanied by the beliefs that love gives meaning to life (90%) [89], having a family is necessary for a happy life (87%) [82], and it is easier to live when in a relationship (55%) [89]. A mere 3% of Poles indicate that they would prefer to live on their own. Among those who are currently single (living alone), most point to being in a close relationship as their ideal living arrangement (aggregated different relationship models: 77%) [92]. Therefore, romantic relationship breakups will likely lead to reevaluating one’s functioning and choices and finding life lessons [77] rather than closing oneself to future romantic endeavors. Poles believe true love is hard to find (70%) but worth the effort [89].

### The present study

The goal of the presented project was to verify how activating a self-story of a broken love (achieved by asking participant to write the story depicting the ended romantic relationship) would affect understanding the past and imagining the future in close relationships. We expected that activation and elaboration of the self-story of broken love would positively influence the quality of thinking on the past and future in this life domain. It would help to better understand the reasons of the past breakup as well as better imagine and project the future of own close relationships. Moreover, we expected that a higher level of narrative plot structuring of the past romantic relationship self-story would increase these effects.

Being led by understanding narrative as a cognitive way of representing reality [5,9,19,53,93] we derive our assumptions from the narrative theory [5,9,13,18,52,94], laboratory research on narrative mindset [34,42] and naturalistic experiments with the self-story activation [20,21,29,30].

By examining the occurrence of narrative thinking in the subjects’ real-life functioning, we aimed to significantly enhance our understanding of the mechanisms and consequences of this cognitive process. To achieve this, we employed a range of observation methods (self-reports, trained coder assessments, and language-use analyses) and diverse types of comparisons to illustrate varying levels of self-story activation. Our focus on the breakup of an informal romantic heterosexual relationship not only expands the field of research but also contributes to the urgent search for effective interventions for individual’s post-breakup [25,27,28]. We begin the study of narrative thinking effects in the area of interpersonal problems in the collaboration with emerging adult women after the romantic breakup in order to address a group for whom this experience might have particular significance in the perspective of the life course.

## Method

### The type of study

This study used a two‐stage naturalistic experimental design (in the form of online surveys) with delayed measurement of experimental manipulation effects and was conducted on research panels from 12.12.2022 to 23.02.2023. The presented methodology and discussed results are part of a broader study on the effects of self-story activation and were selected in accordance with the stated hypotheses.

### Participants

Participants were emerging adult women aged 18–30 who had experienced a breakup from their heterosexual romantic relationship no more than three years prior to the study and had remained single ever since. Further recruitment criteria were informal (non-marital) relationship character and never being married. Suggested although not leading to the exclusion criterion was also current post-breakup suffering.

The rationale for such a homogenous group was the small effect sizes found in previous studies. Sample size estimation was based on a preliminary result of teams’ research (unpublished) in narrative structuring of the breakup of a past romantic relationship, achieving small effect size (*eta*_*p*_^*2*^ = 0.05). Using G*Power 3 [95], with *eta*_*p*_^*2*^ = 0.05 for repeated measures, the ANOVA interaction effect (experimental group and study stage) showed the need for gathering approximately 600 participants for a power level of 85%.

Recruitment was coordinated by the Pollster Research Institute on Polish research panels (Panel Badawczy Reaktor Opinii and Syno MediaPanel). All communication with participants was completed via email. For their participation, women received points they could spend on prizes in the panel shop. Their nominal value was approximately 10 PLN (approximately 2,5 USD; given after participation in both stages), and they were promised a report summarizing their responses and providing insights into the sociocultural context of breakups. Those who did not meet the study basic criteria (age, time since the breakup, relationship status since the breakup, and lack of marriage experience) received consolation points from the research panel.

Exclusion criteria for those who had passed first screening and became study participants were incorrect participation in a research procedure (answering open questions in language other than Polish or partially used English, retaking experimental modification task or taking 11 or more days to participate in stage 2) or not matching detailed research group criteria (being female, having a relationship that ended in breakup – and not in a dissolution due to the partner’s death – [27]; heterosexual character of the relationship).

The Committee of Research Ethics of the Faculty of Psychology at University SWPS in Warsaw approved the study procedure (approval number 2/2023, written consent).

### Measures

### Participant and breakup-related characteristics.

Participants answered questions about their demographic characteristics, current situation, and past romantic relationship.

***Questions from the first stage used to verify match with the study criteria:*** Age – Your year of birth: Please enter it using digits, e.g., 1997. It is necessary to enter the entire year – four digits.

Time since the breakup – How many months have passed since your breakup? Please enter the time since your breakup using numbers. If the time is less than a month, please enter a fraction, e.g., one week – 0.25, two weeks – 0.5.

Relationship status since the breakup – Did you enter into another romantic relationship after this breakup? Yes/No

Marriage experience – Have you ever been married? Yes/No

***Additional questions from the first stage:*** Relationship duration – How many months were you in a relationship with your former partner, with whom you separated? Please enter the duration using numbers.

Role in the breakup – Who made the final decision to end the relationship? Me/Partner/Joint decision/Other situation: which one? Please write your answer in the box below: (space to enter an open answer)

Perceived relationship significance – How important was this terminated relationship to you? Participants responded on a sliding scale (0–100) with the following guidelines: 0 meant Not important, while 100 meant Very important.

Current suffering after breakup: Here and now, how sorry are you for the breakup of this relationship? Participants responded on a sliding scale (0–100), with 0 signifying Not at all and 100 Very.

***Closing questions from the second stage used to verify match with the study criteria:*** Gender – I am: a woman/a man/I identify with another gender/ I prefer not to answer this question

Relationship type – My relationship that ended in a breakup was: heterosexual/homosexual/other - please describe in the box below (space to enter an open answer)

#### Experimental procedure.

The participants were asked to recollect and describe their past romantic relationship. They were randomly divided into two study groups: narrative and control.

In the narrative group, the participants were asked to describe a story depicting the relationship from the start to the end with the breakup.

In the control group, participants were asked to respond to open questions requiring short, concrete answers involving cognitive processes such as listing, identifying, and comparing (fitting into a paradigmatic way of thinking) [5]. Time participants spent on writing their responses was measured.

***Narrative procedure:*** The women were provided with the following instructions:

“We are interested in knowing your individual history of a relationship that ended in a breakup.

We want to ask you to tell it. You will decide what you will share with us and how you will approach telling the story of your former relationship with your partner.

We would like to know how your relationship has changed from getting to know each other through the decision to be together until the breakup.

Please write freely without considering the style or worrying about mistakes. Just as you wrote only for yourself. Most importantly, the description should be true and sincere. You may change your partner’s name and any other details for your comfort. We would be very grateful for longer responses (i.e., more than just one or two sentences). This will allow us to gain a fuller understanding of your experiences, which is particularly important to us.

You can describe the story of your ex-relationship the way one writes book or movie stories. Please do not focus your attention on making it interesting or engaging for the potential reader. The most important thing is that it should be your story. If you do not know how to start, you can use standard formulas: “When we first met.... “, “It all started with...”, or “In the beginning...”.

It usually takes approximately 15 minutes to write such a story. However, you do not have a time limit. When you think you are ready, please start your writing.

Please write your story in the box below:

When you have finished writing, please take a moment to read the entire text. This will enable you to make final corrections and ensure that your story comprehensively and accurately reflects what happened in your ended relationship”.

The full wording of the instruction is available in S2 Appendix, and examples of participants’ responses can be found in S3 Appendix.

***Control procedure:*** Participants responded to 17 open-ended questions related to their broken love. Given that some questions required breaking down the answers into smaller segments, the participants ultimately provided a total of 53 brief responses.

The instructions:

We are interested in learning about your individual experiences, thoughts and assessments related to the relationship that ended in a breakup.

It is up to you to decide what you will share with us and how you will approach completing open-ended tasks.

Please answer freely without caring about the style or worrying about mistakes. It is as if you were doing it just for yourself. The most important thing is to keep your experiences and thoughts real and honest. For your own sense of comfort, you may choose not to provide details or change them to something else.

Example questions:

Listing: Please list the main characteristics of your personality.

You can do this by providing a list of adjectives you would use to describe yourself (e.g., open-minded, warm-hearted, hard-working...) or by listing traits (open-minded, warm-hearted, hard-working...).

Comparing: Please list five similarities between you and your former partner.

These may include habits, traits, preferences, likes and dislikes. If possible, please briefly describe each similarity (in a few words, one sentence at the most).

Identifying: Please sloganize the four most important topics of conflicts that occurred between you.

A complete list of control set tasks, along with example answers, is provided in S1 Appendix.

#### Frequency of thoughts in the last week.

Two questions addressed the frequency of thoughts during the last seven days. Please determine what percentage of your time last week consisted of thoughts about…:

Counterfactual: what would have happened if something in your life and in this relationship had happened differently.

Future-oriented: what your future will be like in close relationships.

Participants responded on a sliding scale (0–100%) with the following guidelines: 0% meant that you did not think about it at all, while 100% meant that these thoughts accompanied you all the time.

#### Event Related Rumination Inventory (ERRI) ([96]; polish adaptation [97]).

The Inventory assesses the frequency of ruminative thoughts connected to a specific life event. Items were adjusted thematically for the situation of a romantic relationship breakup (change from the event/my experience to (this) breakup) and by grammatical gender (to improve their appeal to women). The tool has two ten-item subscales concerning intrusive (e.g., *I thought about this breakup when I did not mean to*) and deliberate thoughts (e.g., *I thought about whether I could find meaning from this breakup*). Participants reported the frequency of experiencing these thoughts on a four-point Likert scale (ranging from 0 [not at all] to 3 [often]). The consistency of scales – Cronbach’s alfa – in a current study (adjusted sample) was excellent, exceeding 0.9 (Intrusive thoughts: 0,95 (first stage, N = 422) – 0,96 (second stage, N = 418); Deliberate thoughts: 0,96 (first stage, N = 422) – 0,93 (second stage, N = 417)).

#### Declared understanding of breakup reasons.

Participants were asked to state their understanding of the reasons for the breakup on a sliding scale (0–100). The question was introduced with the phrase: When I think about why our relationship broke up, and extremes meant 0, I experience a lot of confusion. I don’t understand it. 100 – I fully understand the reasons behind it. The inspiration for the question was Boals et al. ’s [43] findings on the predictive role of subjective understanding of well-being.

#### Description of breakup reasons.

The participants answered an open-ended question about perceived reasons for the breakup:

Why did your relationship end?

Please answer freely, as if you were writing for yourself. Without caring about style or worrying about mistakes. The most important thing is that the thoughts conveyed are genuine and sincere. We ask that you take as much time as you deem necessary to fully present your thoughts.

#### Thoughts on the future in close relationships.

Participants answered the following question about the content of their future-oriented thoughts in area of close relationships

Thoughts on the future in close relationships.

Please share your thoughts over the past week about your future in close relationships.

### Procedure

People declared as women of the required age were invited to participate in a study on romantic relationship breakup and after-functioning. The research began by verifying a match with the study criteria (age, time since the breakup, relationship status since the breakup, and lack of marriage experience).

Participants who met them read the informed consent form as part of the online form and decided whether they agreed to participate in the study. Informed consent was required to proceed with the study. Participants were encouraged to download it in PDF format from the research form to have access to it if needed. The study goal was masked and general transparency was provided for the task content. The participants were informed that they had received only partial information about the study objectives at the start of the study.

After gathering additional data on relationship and participant characteristics (relationship duration, role in the breakup, perceived relationship significance, current suffering after breakup), participants advanced to pretest measurements of the Event Related Ruminations (intrusive and deliberate) (ERRI – [96,97]) and frequency of counterfactual thoughts and future-oriented thoughts about the future in close relationships, both at the time of the last week.

Next step was an assignment to the experimental condition. To support the equivalence of the experimental sets, we used a matched pairs design. Participants were separated into two groups based on their level of Intrusive Event Related Ruminations: those with a level lower than 26 and those with a level equal to or higher than 26. From these groups, they were randomly assigned to the experimental conditions: narrative and control.

All participants responded to questions concerning the past romantic relationship although in different ways. The narrative set participants were asked to write a description of their past relationship, and were guided to do so in the form of a story. Women from the control group answered 17 open-ended questions about their broken love. These tasks ended the first stage.

After about seven days the participants who completed the first stage received an invitation to second stage. Stage 2 had to be completed within less than eleven full days of Stage 1. Participants answered questions on the Event Related Ruminations (intrusive and deliberate) (ERRI – [96,97]), stated declared understanding of breakup reasons, and and frequency of counterfactual thoughts and future-oriented thoughts about the future in close relationships, both during the last week.

After that, participants gave open responses on questions about 1) the breakup reasons and 2) the content of thoughts on the future in close relationships that they had experienced in the previous week.

To prevent discrimination, participants who mismatched the study criteria were allowed to participate; however, their responses were not included in the analysis. At the end of stage two, gender and relationship type were verified.

After the study was completed, the participants received an email disclosing the research goal with an offer to withdraw answers if they did not support it. No such requests were submitted. Regarding the open questions, the participants were informed of their freedom to decide what to share and how to do it.

### Analysis strategies

#### Trained coders assessments.

***General procedure description.*** Scale creation was both theories-based (Narrative Structure Assessment, the Plot Structuring Scale, Coherence of the Description of Breakup Reasons) and data-driven (Theme of thoughts on the future in close relationships). The scale’s applicability to the gathered data was prioritized, as only through this could its usability be guaranteed. Therefore, the scales (except for the full version of the Plot Structuring Scale) were created using the study’s answer sample and referred to participant’s responses as illustrations of the scale’s categories. Detailed scale descriptions are presented in the S1-S5 Appendixes.

The trained coders participated in the training before the assessment began. They read the scoring instructions, asked questions, and provided suggestions for improvement. Subsequently, they worked independently during the training period. The scores were then matched, and discrepancies were discussed. The instructions were adjusted until a shared understanding and convergence of the evaluations were achieved. After obtaining satisfactory scoring reliability during the training, proper analyses were performed. Trained coders worked in pairs and scored the responses separately. In the following step, they met to gain consensus on disparate assessments. They were blinded to the study conditions and the detailed hypotheses. During the study, six trained coders worked with the study materials (in various pairings, stable within one set of analyses). Inter-rater reliability (IRR) was calculated using the interclass correlation (*ICC*) for scales (model: two-way mixed, type: absolute agreement, interclass correlation coefficient: average measures) [98] and kappa (κ) for categorical analyses (presence of narrative structure, content of future thoughts) [99].

***Stage 1: Participation quality.*** Narrative structuring is instinctual, while a non-automatic cognitive process which presence requires verification before connecting it with the observed effects. The most objectively observable sign is the presentation of a self-story (told narrative) with signs of a canonical story structure.

At the same time, it is essential to evaluate the level of effort put into the control condition to determine whether participants in that group devoted as much thought to previous relationships as those in the narrative condition and, therefore, differed only in the structure of their thought processes (paradigmatic vs. narrative).

Control Set – Answers Completion

This scoring was not preceded by training because of the simple format of the data cleaning. Trained coders read the participants’ answers and, acting on instructions, decided whether each answer could be viewed as a likely answer to the posed question. Unmatching answers (such as filling answer boxes with nonsensical words, punctuation marks, and repetitions) were cleared, allowing percentage completion calculation. The interrater reliability (IRR) assessed by interclass correlation (ICC) was excellent:.999 (.998−.999; *p* < .001; N = 168/ N = 169) [100,101].

A detailed description of how the data were processed, along with sample responses from the participants, is provided in S1 Appendix.

Narrative Set – Narrative Structure Assessment

Narrative Structure Assessment is part of the Plot Structuring Scale, determining the difference between scores of 0 (lack of narrative structure) and at least 1 (incomplete story structure). Scale construction was based on the classical narrative definition, focusing on the presence of the actor’s intention, complication, and plot (with cause-effect and temporal way of connecting events) [5,6,52]. An additional requirement for discerning the presence of narrative structure is depicting the entire duration of a relationship (not chosen episode) [14,102]. Full trained coders instruction can be seen in S2 Appendix while the example responses are presented in S3 Appendix (non-narrative responses are scored 0, narrative have scores 1–4).

Trained coders scored all participants’ narratives, which were prognosticated for the presence of a narrative structure and came from participants who took part in stage 2. Empty answers, the use of nonsensical words, etc., were not given to trained coders.

In the first scoring within training process (N = 10), average coders IRR was moderate κ = .58 (pair 1: κ = .60; *p* = .038; pair 2: κ = .55; *p* = .053) [101]. In the second stage (N = 30), the average IRR was substantial κ = .63 (pair 1: κ = .75; *p* < .001; pair 2: κ = .51; *p* = .004). Substantial agreement allowed the start of the main scoring. Scores from the second scoring phase were accepted as final, with 11 scores being agreed upon at the coders’ and leaders’ meeting. Main analyses (N = 251, divided between two coders pairs) had substantial IRR of κ = .68 (pair 1: κ = .77; *p* < .001, N = 126; pair 2: κ = .60; *p* < .001; N = 125). Additionally, to guarantee reliability in the coders’ understanding of the presence of a narrative structure, each pair assessed 60 answers. IRR of coders pairs ranged between.60−.86 (*p* < .001). Each classification disagreement was discussed within each coder’s responsible pair, and a final consensus score was provided (pair 1: N = 12, 9.52% of discrepant classifications; pair 2: N = 22, 17.6% of discrepant classifications).

***The Plot Structuring Scale.*** The Plot Structuring Scale assesses the degree to which the presented text has a narrative structure and content adequate for the indicated narrative theme. An answer with a high level of plot structuring depicts the process of formation and disintegration of relationships using narrative reasoning, that is, referring to showing the intentions of the characters, the complications along the way of their realization, and the consequences of the actions taken to illustrate this with specific events and actions, and the context of consciousness accompanying the characters.

The scale has five levels, from which a score of 0 defines a lack of narrative structure and scores between 1 and 4 characterize the plot structuring quality (see Table 1). Narrative Structure Assessment (presented higher) describes differentiating scores of 0 from all others. Whenever a one-point discrepancy occurred (in scores of 1–4), the final score was the average score. In case of scoring discrepancies between two or more scores, participants’ responses were sent back to the trained coding pair, and their final scores were determined through a discussion between the coders.

**Table 1:** Definitions of the plot structuring levels for the trained coders.

Score	Definition
**0**	The structure of the description does not allow the text to be considered a narrative (critical deficiencies in meeting the requirements for the presence of a narrative structure).
**1**	The description meets the basic requirements of the narrative structure, however, the narrative remains at least partially incomprehensible (e.g., vagueness of the intentions of the characters and the complications they encounter, disordered linking of events – chronological and/or cause and effect) or fragmentary (e.g., deficiencies in the presentation of the introduction, development or completion of the narrative flow with a completed link, the transmission of too little meaningful information, the transmission of off-topic content).
**2**	The description meets the requirements for the presence of a narrative structure, with an introduction, development and conclusion. The way in which events are tied together is generally good. Intention, complication and their interaction are presented, although they are not elaborated, or despite their good presentation, the description often deviates from the main theme, so that it contains a poorly outlined narrative thread. Divergence from the theme can be, for example, in the form of extended reflections that disrupt the narrative plot (sequential presentation of events).
**3**	The description meets the requirements for the presence of a narrative structure, with an introduction, development and conclusion. The way in which events are tied together is good; the chronological and/or cause-and-effect relationships between events and/or episodes are understandable. Intention, complication and their interaction are presented in a good way, creating a well-defined plot. The narrative elements described are generally on topic, and if deviating from it, do not hinder the reception of the narrative.
**4**	The description fully realizes the assumptions of the presence of a narrative structure and contains an articulated plot. Intention, complication and their interaction are presented in a broad way. The way in which events are tied together is very good – the chronological and/or cause-and-effect connections between events are clear. The narrative elements described are related to the main plot and maintain reasonable areas of volume (not too short and not too long). The process of change that the characters and/or their relationship undergo is presented while maintaining the distinct state of knowledge/awareness realistically available to the characters at the given stages of the relationship (knowledge of the ending does not determine the course of the presented story). Elements/events of the narrative have been well explained (this can be achieved, for example, through a broad representation of the area of consciousness accompanying the heroine/heroes).

The full text of the instructions for trained coders, along with examples of participants’ responses corresponding to each level, can be found at S3 Appendix.

The scale was used in a previous team study with a similar methodology, and trained coders had experience in its use. One coder did not participate in the first study scoring and received individual training from the first author, assessing narrative responses whose scores were agreed upon during the training period from another study. Her IRR *ICC* was between.90−.93 (scoring session 1: ICC (10) =.90; *p* < .001, CI 95% (.62;.97) (N = 11); scoring session 2: ICC (14) =.93; *p* < .001, CI 95% (.78;.98) (N = 15). Before the main scoring, the initial IRR was verified using 30 random participant responses. Among the five trained coders, one had a lower IRR than the others, and was excluded from further scoring. The IRR of the four chosen trained coders was at an average ICC of.92 (.87−.97; *p* < .001, degrees of freedom: 29, N = 30). Nineteen response scores were accepted because their scores were identical between coders in calibration session. The remaining 11 were referred to for post-renewal analysis within the trained coder pairs.

Pairs were determined by maximizing the IRR of the trained coders on a calibration scoring basis. Each pair received 97 participants’ responses to the ratings (194 responses in the entire study). IRR of main assessment average were excellent ICC = 0.91 (0.88–0.94; p < 0.001, degrees of freedom: 96) (pair of judges 1: ICC (96) = 0.88; *p* < 0.001, CI 95% (0.77; 0.93); pair of judges 2: ICC (96) = 0.94; *p* < 0.001, CI 95% (0.91; 0.96)) [99,100].

Four answers that received scores discrepant by two or more points were sent for coders’ pair discussions and consensual decisions on final points.

***Stage 2: Measurement of narrative framing effects. Coherence of the Description of Breakup Reasons.*** This scale was used to assess the coherence of answers to an open question Description of Breakup Reasons.

Coherence encompasses the organizational arrangement of information within an expression as well as the recipient’s ease of deciphering its intended meaning [103]. A coherent text is characterized by interconnected components that harmonize without contradiction, aligning seamlessly with the overarching context and thematic framework of the utterance (adapted definition based on Reinhart, 1980; as cited in [103]).

The scale has a 5-point Likert scale: description is characterized by.... coherence of argumentation. Scores: 1, absent; 2, very low; 3, sufficient; 4, good; and 5, very good. A point of six marked answers not suitable for scoring (e.g., incomprehensible, containing a refusal to answer).

The complete set of instructions for trained coders, along with examples of participants’ responses is available at S4 Appendix.

The final IRR at the end of the training period was ICC (29) =.82; *p* < .001; CI 95% (.61;.91). All responses were returned to the pool and were evaluated using a primary scoring system. Trained coders assessed 653 responses, of which 646 were available for study analysis (exclusions due to critical flaws in survey participation). The IRR in the main scoring for proper responses was excellent [100]: ICC (645) =.79; *p* < .001; CI 95% (.75;.82). Eighty-five responses (out of 653) were returned to trained coders because of discrepancies of two points or higher or inconsistent qualifications of the answer as unsuitable for evaluation. Final scores were determined by consensus through a discussion between the coders. In the case of a point discrepancy, the final score was the average of the trained coders’ scores.

***The theme of thoughts on the future in close relationships.*** The scale was used for the thematic categorization of answers to open question Thoughts on the future in close relationships.

Using an inductive approach based on lecture of the study answers and discussions with trained coders, ten categories were identified and grouped into four main categories.

1) Interest in thinking about the future: This description presents ideas of a possible future or plans about the future (subcategories: focus on personal intentions, neutral future thoughts, focus on the problem).
2) Reluctance to think about the future (subcategories: no thoughts on close relationships, lack of interest in romantic relationships, and anchoring in the past).
3) Imagined helplessness in forming close relationships (subcategory: helplessness)
4) Other (subcategories: mixed – more than one category, other, not suitable for assessment)

The full set of guidelines for trained coders, as well as examples of participants’ answers, can be accessed at S5 Appendix.

In total, 631 assessments were performed by trained coders, of which 625 were based on data on proper structural properties, allowing for study inclusion. The IRR was calculated for the main categories (allowing for differences within the mixed category). The IRR after training was κ = .51, *p* < .001 (N = 30) (moderate agreement – [101]). All responses were returned to the pool for scoring by independently trained coders. Next, trained coders scored 100 participants’ responses, receiving a substantial agreement of κ = 0.62, p < 0.001 (N = 100). Subsequently, an additional calibration training was performed. The main scoring consisted of 531 responses with substantial agreement (κ = .71, *p* < .001). The IRR of trained coders in both scoring rounds was κ = .70, *p* < .001 (N = 625) (for the main categories; both rounds of evaluations combined, counted in the program, not averaged). All discrepancies (*N* = 140; 19 reconciled jointly with the project leader after round 1; for 631 evaluations) were reconciled with trained coders discussing the final score during the meeting.

To compare interest and reluctance to think about the future, answers initially classified as mixed but fully fitting one main category were included in either the “interest in future thinking” or “reluctance in future thinking” category.

#### Language analyses of the open texts provided by the subjects.

The Linguistic Inquiry and Word Count (LIWC) program [48] calculates words within specified categories ranging from linguistic dimensions through Psychological Processes, Relativity ending with thematic categories of Personal Concerns. In most cases, the results showed the proportion of words in the entire text (not applicable to word counts and words per sentence). In the current study, version 2001 was used as the only version with a Polish dictionary [49]. Another linguistic program specializing in the Polish language, the Literary Exploration Machine (LEM, from CLARIN PL infrastructure), was chosen to analyze verb forms [50], allowing detailed analyses of verbs in their grammatical types, modes, and tense forms. The program calculates the number of verbs of a particular type. Responses exhibiting a dispersion exceeding an interquartile range (IQR) of 2.2 [104] for word count were excluded from the analyses. For the description of the reasons for breakup, the threshold was set at < 131.4 words for the group comparison (N = 382, N_n_ = 186, N_c_ = 196; excluded: N = 25) and for comparisons within the narrative condition (N = 292, N_n_ = 186, N_nc_ = 106; excluded: N = 13) while for analyses of groups with different levels of plot structuring (narrating participants from the narrative condition) at < 151,4 (N = 191; excluded: N = 8). For thoughts on the future in close relationships, the accepted word count was < 78.4 for group comparison (N = 385, N_n_ = 190, N_c_ = 195; excluded: N = 15), and < 78.2, for comparisons within the narrative condition (N = 283, N_n_ = 190, N_nc_ = 93; excluded: N = 13), while for analyses of groups with different levels of plot structuring (narrating participants from the narrative condition) it was < 87 (N = 195; excluded: N = 6). Due to the non-normal distribution of most variables (high kurtosis and skewness), which is normal for language variables [47] the nonparametric tests of Mann-Whitney U and the Kruskal-Wallis test with an effect size of epsilon squared [105] and Bonferroni correction for significance. From the exploratory analyses, only variables with high potential for worthwhile psychological interpretation were reported.

#### Analysis of potential factors preventing narrative structure classification.

An exploratory, post hoc analysis was conducted to provide insights into possible reasons why trained coders decided to exclude participants from the narrative set. The first manuscript author, working in line with postulates of the thematic analysis [106], classified non-narrative responses depicting the most important characteristics that could exclude a response from being classified as a self-story. After it was done, a pair of trained coders were asked to help classify problematic responses and reduce the number of categories during shared discussion. After the final clarification session conducted by the first author final identified meta categories were as follows:

1. Failure to answer the question: leaving the response empty, submitting nonsensical words or interpunction marks; avoiding answering the question – declaring outright refusal to answer or not remembering the history of the relationship; given response does not provide any knowledge about the relationship – answer truncated, possible technical error in completing the survey.
2. Conveying a small amount of information about the relationship in a non-narrative and non-argumentative manner: fragmentary package of information about the relationship and/or breakup. Neither has well-defined narrative features nor does it provide a good argument/explanation of the reasons for the relationship’s breakup.
3. Justification for the breakup of the relationship - opinion/reflection/argumentation: It may have elements of chronology but it is mostly a presentation of opinions, reflections or arguments connected with the breakup of the relationship.
4. Chronological presentation of events, with an absence of the narrative thread: A chronologically ordered report of the events of the time of the relationship. It lacks the narrative thread (outlining the characters’ intentions, complications, and interaction).
5. Partial narrative – presents in a narrative way only selected stages of the relationship (getting to know each other/entering the relationship, time of the relationship, deterioration, breakup). It can refer to multiple stages; however, how it does so does not provide continuity in the narrative thread. It differs from reflection/argumentation by outlining chronology and using narrative logic for description – i.e., it refers to the presenting specific events, along with their consequences, and shows the intentions of the parties involved. In the case of more than one episode presentation, the exclusion criteria may be a lack of a clear description of why the relationship broke up/how the breakup occurred.
6. Narrative not on topic – a text with a narrative structure or a structure close to it, but it deals with a different topic than the breakup of a personal romantic relationship (the breakup of a relationship is a side plot of the narrative).

## Results

The first part provides information on study participants, including data on study participation, general characteristics, equivalence of conditions, and plot-structuring of participants’ self-stories. Next, the effects of self-story activation are presented, including analyses based on comparisons of conditions (narrative vs. control), sharing a self-story (within the narrative group: narrating vs. non-narrating participants), and connections between the plot structuring level and the dependent variables. The description of the results concludes with exploratory qualitative assessments of potential reasons for not recognizing the narrative structure in some responses of the narrative set participants to the task of writing the self-story of broken love (experimental modification task, stage 1). The analyses were conducted using IBM SPSS Statistics (version 28 and 29).

### Participants

#### General data on participation and condition equivalence.

**Participation analysis:** After verification of the criteria fulfillment and subsequent informed consent, 925 participants began the study, of whom 913 completed it. Stage 2 was approached by 684 people, of whom 668 completed it. The dropout rates for the raw data were 25.08% (to start Stage 2) and 26.83% (to complete Stage 2). Additional analyses of the content provided by the participants to verify the structural quality of participation indicated additional reasons for not fitting into the criteria, consisting of the breakup of a relationship due to the death of a partner (four people), declaration of no relationship (1), other declared gender than female (5), and separation from a relationship type other than heterosexual (22 homosexual and one time other) – 33 people. Furthermore, one person submitted answers to open-ended questions in language other than Polish or English. Six people participated in Stage 1 twice, so they were excluded because of the disruption of the experimental modification experience. Hence, the correct structural approach to Stage 1 was characterized by 873 people. Of these, 645 approached stage 2. Of these, 14 were excluded because they took too long to reach Stage 2 (time to join not less than eleven days) (631). Fifteen people did not complete Stage 2, resulting in 616 participants who completed Stage 2 with structurally correct participation. Dropouts in terms of structurally correct data were 27.72% (to start Stage 2) and 29.44% (to complete Stage 2).

Participants recruited from different research panels: Panel Badawczy Reaktor Opinii (N = 721) and Syno MediaPanel (N = 138) were equal in participant and breakup-related characteristics (age, time since the breakup, relationship duration, role in the breakup, perceived relationship significance, current suffering after breakup) and cognitive functioning in stage 1 (Event Related Ruminations (intrusive and deliberate); frequency of future-oriented thoughts the last week). They, however, differed in frequency of counterfactual thoughts in the last week (*z* = 2.85, *p* = .004, *r* = .10), with participants from the smaller group (Syno MediaPanel) declaring their higher level (Me = 57.00; IQR (Q1–Q3) = 46.50 (29–75.5)) than those from the bigger group (Panel Badawczy Reaktor Opinii) (Me = 44.00; IQR (Q1–Q3) = 54 (16–70)). Due to the small number of differences, it was decided to continue to consider participants from both research panels together.

**Verification of participation in experimental manipulation tasks:** Responses from participants with structurally correct data who started at least second stage were assessed for participation quality (see Methods for details). In the narrative group of 322 participants, 204 (202 completed the entire study) fulfilled all criteria and 118 were excluded due to a lack of self-story creation (exclusions: 36.65%). In the control group, from 309 participants, 218 were accepted (209 completed the whole study) and 91 were excluded due to a completion level lower than ≥ 74.9% (exclusions: 29.45%). Making an allowance for the fact that a self-story was created (and control group participants were adequately engaged) is necessary to enable the accurate attribution of intervention effects to narrative thinking.

**Participants characteristics:** Participants considered in the main analyses were 422 emerging adult women (18–30 years old), on average less than a year after the breakup of an informal romantic relationship that lasted on average almost two years, who saw their past relationship as significant and currently declare moderate suffering after breakup. Detailed characteristics are presented in Table 2., while the role participants played in their breakup is to be found in Table 3. The time since participation in stages 1–2 was on average 7.33 days (SD = 1.01, Min = 6.08, Max = 10.96).

**Table 2:** Descriptive statistics of participant and breakup-related characteristics for quantitative variables.

Variable	N	M	SD	Me	Min	Max
Age (years)	422	23.55	3.28	23	18	30
Relationship duration*	420*	22.20	23.15	14	1	206
Time since the breakup (months)**	422	10.16	8.80	8	0,04	36
Current suffering after breakup (0–100)	422	51.53	32.07	52	0	100
Perceived relationship significance (0–100)	422	82.22	18.65	85	0	100

*Participants declaring relationships longer than 240 months were excluded from this analysis.

** A data entry error was identified for one participant who reported a negative time since the breakup (−0.5 months). This value was adjusted to 0.5 months.

**Table 3:** Participant’s declared role in the breakup.

Breakup Role	N	Percentage
Initiator	196	46.4
Recipient	114	27.0
Mutual decision	101	23.9
Other	11	2.6

**Intergroup differences between experimental conditions:** Within the cleared data, there were no differences between participants in participant and breakup-related characteristics, except for current suffering after breakup (*t*(406.85) = −2.15; *p* = .032; CI 95% (−12.84, −0.56); *d* = −0.21 CI 95% *d* (−0.40, −0.02). Two-sided significance). Those in the narrative condition reported lower levels (M_n_ = 48.07; SD_n _= 33.76; N_n_ = 204) than the control group participants (M_c _= 54.77; SD_c_ = 30.12; N_c_ = 218). This difference can be attributed to the data cleaning procedure, as such a difference did not occur in the basic data (in participants who started second stage) (*t*(628.69) = −1.65; *p* = .100; CI 95% (−9.44;.83); *d* = −.13 CI 95% *d* (−.29;.03). Two-sided significance).

When it comes to pretest variables connected with cognitive processes, groups were equal in Event Related Ruminations (intrusive and deliberate) and frequency of future-oriented thoughts. However, they differed in frequency of counterfactual thoughts in the last week (*t*(417.39) = −2.03; *p* = .043; CI 95% (−11.44; −.18); *d* = −.20 CI 95% *d* (−.39; −.01). Two-sided significance). Women who created a self-story were characterized by their lower frequency (M_n_ = 41.46; SD_n_ = 29.59; N_n_ = 204) than control group participants (M_c_ = 47.27; SD_c_ = 29.22; N_c_ = 218). Once more, this difference was not observed in the basic data (*t*(627.94) = −1.28; *p* = .200; CI 95% (−7.90; 1.66); *d* = −.10 CI 95% *d* (−.26;.05). Two-sided significance), therefore should be connected to the used the data-cleaning procedure.

Analysis of a time spent on doing the experimental modification task was assessed by Mann-Whitney U test because of severe kurtosis and skewedness. The experimental conditions differed in time they dedicated to their respective activities (*z* = 3.02, *p* = .003, *r* = .15) with control group spending more time (in minutes) (Me = 15.31; IQR (Q1–Q3) = 10.12 (10.97–21.10)) than narrative participants (Me = 12.48; IQR (Q1–Q3) = 15.21 (7.15–22.37)). The same effect pattern was present on the basic data, so its occurrence is not connected with the data-cleaning procedure (*z* = 6.83, *p* < 0.001, *r* = .27) (control set: Me = 12.86; IQR (Q1–Q3) = 10.10 (8.93–19.03); narrative set: Me = 7.74; IQR (Q1–Q3) = 13.53 (3.67–17.21)).

Current suffering after breakup, frequency of counterfactual thoughts in the last week and the time of engagement in the experimental modification tasks measured at the first stage were included as covariates in the selected analyses comparing the narrative and control conditions.

#### The narrative set participants.

The narrative group that started Stage 2 (N = 322) comprised women with different levels of plot structuring of the broken love description. For easier inter-subgroup comparison, five groups of plot structuring quality were created: lack of a narrative structure characterizing non-narrating participants (score 0, N = 118, 36.6% of the group) and four subgroups within narrating participants (N = 204): incomplete (score 1, N = 63, 19.6%), basic (score 1,5−2, N = 69, 21.4%), good (score 2,5−3, N = 46, 14.3%), and excellent narrative structure (score 3,5−4, N = 26, 8.1%).

In subsequent analyses, the number within the group may vary because of dropping out before reaching each measurement, or not providing a response possible for the assessment. More than one-third of participants in the narrative group failed to narrate their experiences. This disproportion (approximately 1:2) is the reason for utilizing the nonparametric Mann-Whitney U test in most of the subsequent comparisons. The narrative structuring mean (M) was 1.30, with a standard deviation (SD) of 1.26, ranging from a minimum value of 0 to a maximum value of 4; the distribution showed a skewness of.61 and a kurtosis of −.68.

**Differences between narrating and non-narrating participants:** There were no differences between the groups in pretest variables: participant and breakup-related characteristics (age, time since the breakup, relationship duration, role in the breakup, current suffering after breakup) and cognitive functioning in stage 1 (Event Related Ruminations (intrusive and deliberate); frequency of counterfactual and future-oriented thoughts the last week).

The only difference between narrating and non-narrating participants in the psychological variables was the perceived relationship significance (*z* = −2.37, *p* = .018, *r* = .13), with those women who created self-stories seeing it as more important (N = 204; Me = 85.00; IQR (Q1–Q3) = 28.75 (71.25–100)) than those who did not provide a narrative description (N = 118; Me = 80.00; IQR (Q1–Q3) = 35 (64–99)).

Additionally, the narrating participants spent more time writing their texts (Me = 12.48; IQR (Q1–Q3) = 15.21 (7.15–22.37)) than did those whose responses were not qualified as self-stories (Me = 2.34; IQR (Q1–Q3) = 4.95 (0.83–5.77)) (*z* = −11.43, *p* < .001, *r* = .64).

When possible, these differences (perceived relationship significance and the time of engagement in the experimental modification task) were controlled for by presenting ANCOVA supporting nonparametric test results.

**Differences between narrating participants of different levels of self-story’s plot structuring:** There were no differences between the four narrating subgroups in pretest variables: participant and breakup-related characteristics and cognitive functioning in stage 1.

The narrating subgroups differed in the time they spent on writing their self-story (*H*(3) = 83.67; *p* < .001; ∊^2^ = 0.41; *N* = 204) with the clear pattern of growing time between those who created incomplete (*Me* = 6.66; IQR (Q1–Q3) = 5.73 (4.88–10.61)) (p < 0.001 in comparison with all other groups), basic (Me = 11.67; IQR (Q1–Q3) = 11.97 (7.37–19.35)) (p = .004 in comparison with good structure and p < 0.001 in comparison with excellent structure) and good narrative structure good (Me = 18.21; IQR (Q1–Q3) = 14.52 (12.64–27.16)), with no difference in time (p = .235) needed to write good or excellent (Me = 30.92; IQR (Q1–Q3) = 25.20 (19.47–44.67)) narrative structure. Significance levels were adjusted using the Bonferroni method.

### The effects of a self-story activation

In the two sets of comparisons, we compared groups with different levels of self-story activation. Firstly, the study conditions will be compared to verify the experimental self-story activation (the narrative group) with a control condition task designed to prevent or minimize self-story activation. The second comparison of self-story activation levels will be within the narrative condition between those who shared a self-story and those who failed to do so despite explicit instruction, signifying the difference between willingness for self-story activation and inability or resistance to narrative thinking or sharing its effects. The final section presents the connections between the plot structuring of a self-story of broken love and the strength of the observed effects.

#### Participants after the narrative framing will have activated more beneficial cognitive processes revealed in language use.

In subsequent analyses, due to the use of the Mann-Whitney U test, baseline differences between the narrative and control conditions (current suffering after breakup, frequency of counterfactual thoughts in the last week and the time of engagement in the experimental modification tasks) and between narrating and non-narrating participants (perceived relationship significance, the time of engagement in the experimental modification tasks) could not be controlled. The analysis of language-related variables suggests use of non-parametric tests, as the distribution of words within specific categories may deviate from normal distribution [47].

The programs used for the analyses were The Linguistic Inquiry and Word Count (LIWC) program [48,49] and the Literary Exploration Machine (LEM, from CLARIN PL infrastructure) [50].

Analyses showed that in both comparisons (between the conditions and within the narrative group), participants who created self-stories wrote reflections (both tasks) that had a higher word count (WC – LIWC), a greater proportion of words showing causation (Cause, e.g., because, effect, hence – LIWC), and a higher number of used verbs (Verb – LEM). Breakup reasons written by narrative participants had more past tense verbs, whereas their thoughts on the future in close relationships used more future verbs (LEM).

Exploratory analyses also showed that a higher number of exclusive words (e.g., but, except, without – LIWC) being part of the space category, showing the orientation of objects in space and a higher presence of present tense verbs and first-person singular verbs (LEM).

Additional exploratory analyses of **the breakup reasons descriptions** showed a higher use of Insight (e.g., think, know, consider – LIWC) and Tentative (e.g., maybe, perhaps, guess – LIWC) words. Participants used more 1st person singular pronouns (I – LIWC). The thematic scope of the answers featured more words connected with occupation (e.g., work, class, boss – LIWC) and physical states and functions (Physcal) (e.g., ache, breast, sleep – LIWC).

Only in the condition comparison, higher use of certainty and cognitive processes words and fewer words expressing anger were seen in the narrative group participants (LIWC).

Worth noting from the exploration of **thoughts on the future in close relationships** are condition differences in Motion words (e.g., walk, move, go – LIWC) with higher levels in the narrative group. Also – at the statistical tendency – higher presence of Cognitive processes words in control group participants (LIWC).

Within the narrative condition, participants who created self-stories used more words asserting certainty (e.g., always, never) and showed higher social orientation in thematic analysis (Social: e.g., talk, us, friend, in subcategories: Human and Other references to people) and higher pronoun use (Total pronouns including Total third-person pronouns) (LIWC).

Detailed statistics for the breakup reasons descriptions are presented in Table 4, and for thoughts on the future in close relationships in Table 5.

**Table 4:** Statistics of language use comparison within study conditions and within narrative group in task of the Description of Breakup Reasons.

Analysis type	Variable	Presence	The narrative group (*N* = 186)			The control group (*N* = 196)			Parameters of the test statistic (*WC* < 131.4)							Narrating participants (*N* = 186)			Non-narrating participants (*N* = 106)			Parameters of the test statistic (*WC* < 131.4)						
			Me	IQR	Usage percentage	Me	IQR	Usage percentage	*U*	*z*	*p*	*r*	Range		Higher leven in group	Me	IQR	Usage percentage	Me	IQR	Usage percentage	*U*	*z*	*p*	*r*	Range		Higher leven in group
													The narrative group	The control group												Narrating participants	Non-narrating participants	
Hypotheses testing	Word Count (WC)^1^	All responses and comparissons	30.00	34.25	100.00	19.00	26.00	100.00	13497.50	−4.39	<.001***	.22	216.93	167.36	N	30.00	34.25	100.00	16.00	27.00	100.00	6526.00	−4.80	<.001***	.28	164.41	115.07	N
	Causation (Cause)^1^	All responses and comparissons	2.86	5.43	64.00	.00	2.96	38.80	13289,50	−4.87	<.001***	.25	218.05	166.30	N	2.86	5.43	64.00	.00	4.04	43.40	7754.00	−3.17	.002**	.19	157.81	126.65	N
	Verb^2^	All responses and comparissons	6.00	8.00	96.80	4.00	6.00	88.80	14018.00	−3.92	<.001***	.20	214.13	170.02	N	6.00	8.00	96.80	3.00	6.00	86.80	6899.50	−4.28	<.001***	.25	162.41	118.59	N
	Past tense verb^2^	Breakup reasons description	3.00	4.00	95.20	2.00	4.00	85.70	14351.50	−3.62	<.001***	.19	212.34	171.72	N	3.00	4.00	95.20	2.00	3.00	82.10	6655.00	−4.65	<.001***	.27	163.72	116.28	N
Exploration	1st person singular verb^2^	All responses and comparissons	1.00	3.00	62.90	.00	2.00	39.30	14515.00	−3.68	<.001***	.19	211.46	172.56	N	1.00	3.00	62.90	.00	2.00	41.50	7844.50	−3.06	.002**	.18	157.33	127.50	N
	Present tense verb^2^	All responses and comparissons	1.00	2.00	53.20	.00	1.00	35.70	15310.00	−2.99	.003**	.15	207.19	176.61	N	1.00	2.00	53.20	.00	1.00	41.50	8569.00	−2.01	.044*	.12	153.43	134.34	N
	Exclusive (Excl)^1^	All responses and comparissons	.00	1.80	33.90	.00	.00	18.40	15585.00	−3.18	.001**	.16	205.71	178.02	N	.00	1.80	33.90	.00	.00	17.90	8369.00	−2.71	.007**	.16	154.51	132.45	N
	1st person singular pronouns (I)^1^	Breakup reasons description	3.23	6.06	65.60	.00	5.56	46.90	15701.00	−2.45	.014*	.13	205.09	178.61	N	3.23	6.06	65.60	.00	5.30	43.40	8196.00	−2.49	.013*	.15	155.44	130.82	N
	Insight (Insight)^1^	Breakup reasons description	6.06	6.77	86.60	4.13	7.84	63.80	14086.50	−3.87	<.001***	.20	213.77	170.37	N	6.06	6.77	86.60	4.55	9.16	63.20	8363.00	−2.17	.030*	.13	154.54	132.40	N
	Tentative (Tentat)^1^	Breakup reasons description	1.36	4.00	52.70	.00	3.22	37.80	15688.00	−2.58	.010*	.13	205.16	178.54	N	1.36	4.00	52.70	.00	2.32	33.00	8013.00	−2.90	.004**	.17	156.42	129.09	N
	Occupation (Occup)^1^	Breakup reasons description	.00	2.90	39.20	.00	.00	23.50	15270.50	−3.34	.001**	.17	207.40	176.41	N	.00	2.90	39.20	.00	.34	24.50	8628.50	−2.10	.036*	.12	153.11	134.90	N
	Physical states and functions (Physcal)^1^	Breakup reasons description	.00	.99	25.80	.00	.00	16.80	16627.00	−2.08	.038*	.11	200.11	183.33	N	.00	.99	25.80	.00	.00	15.10	8841.00	−2.03	.043*	.12	151.97	136.91	N
	Certainty (Certain)^1^	In both tasks, but in different comparisons	.00	2.31	36.60	.00	.00	23.00	15855.00	−2.73	.006**	.14	204.26	179.39	N													
	Anger (Anger)^1^	Breakup reasons description: only condition comparison	.00	1.15	27.40	.00	3.49	36.70	20419.50	2.45	.014*	.13	179.72	202.68	C													
	Cognitive Processes (Cogmech)^1^	Condition comparissons in both open tasks (varying effect directions and significance across tasks)	17.21	9.22	98.40	14.50	13.46	86.20	15089.50	−2.91	.004**	.15	208.37	175.49	N													

Note: *p < .05, **p < .01, ***p < .001.

^1^Variable from the Linguistic Inquiry and Word Count (LIWC) program [48,49], ^2^ Variable from Literary Exploration Machine (LEM, [50]).

Usage percentage: Percentage of responses containing at least one word from the category.

**Table 5:** Statistics of language use comparison within study conditions and within narrative group in task of recalling Thoughts on the future in close relationships.

Analysis type	Variable	Presence	The narrative group (*N* = 190)			The control group (*N* = 195)			Parameters of the test statistic (*WC* < 78.4)							Narrating participants (*N* = 190)			Non-narrating participants (*N* = 93)			Parameters of the test statistic (*WC* < 78.2)						
			*Me*	*IQR*	Usage percentage	*Me*	*IQR*	Usage percentage	*U*	z	*p*	*r*	Range		Higher leven in group	*Me*	*IQR*	Usage percentage	*Me*	*IQR*	Usage percentage	*U*	*z*	*p*	*r*	Range		Higher leven in group
													The narrative group	The control group												Narrating participants	Non-narrating participants	
Hypotheses testing	Word Count (WC)^1^	All responses and comparissons	23.00	20.25	100.00	15.00	17.00	100.00	13360.00	−4.73	<.001***	.24	220.18	166.51	N	23.00	20.25	100.00	13.00	17.50	100.00	5366.50	−5.37	<.001***	.32	160.26	104.70	N
	Causation (Cause)^1^	All responses and comparissons	.00	4.00	43.70	.00	2.08	27.20	15899.00	−2.82	.005**	.14	206.82	179.53	N	.00	4.00	43.70	.00	.00	22.60	7268.00	−2.80	.005**	.17	150.25	125.15	N
	Verb^2^	All responses and comparissons	5.00	4.00	98.40	3.00	4.00	95.40	13819.00	−4.34	<.001***	.22	217.77	168.87	N	5.00	4.00	98.40	3.00	4.00	89.20	5629.50	−4.99	<.001***	.30	158.87	107.53	N
	Future tense verb^2^	Thoughts on the future in close relationships	1.00	2.00	65.80	1.00	2.00	59.50	15799.00	−2.60	.009**	.13	207.35	179.02	N	1.00	2.00	65.80	1.00	1.50	51.60	6870.50	−3.17	.002**	.19	152.34	120.88	N
Exploration	1st person singular verb^2^	All responses and comparissons	2.00	3.00	93.20	2.00	2.00	82.60	13855.50	−4.36	<.001***	.22	217.58	169.05	N	2.00	3.00	93.20	2.00	1.00	79.60	5892.00	−4.63	<.001***	.28	157.49	110.35	N
	Present tense verb^2^	All responses and comparissons	1.00	3.00	71.10	1.00	2.00	62.10	16195.50	−2.20	.027*	.11	205.26	181.05	N	1.00	3.00	71.10	1.00	2.00	59.10	7044.50	−2.87	.004**	.17	151.42	122.75	N
	Exclusive (Excl)^1^	All responses and comparissons	.00	.00	21.60	.00	.00	11.80	16700.50	−2.58	.010*	.13	202.60	183.64	N	.00	.00	21.60	.00	.00	10.80	7923.50	−2.10	.035*	.12	146.80	132.20	N
	Cognitive Processes (Cogmech)^1^	Condition comparissons in both open tasks (varying effect directions and significance across tasks)	19.27	10.71	95.80	21.43	13.19	93.80	20662.50	1.96	.050	.10	181.75	203.96	C													
	Motion^1^	Thoughts on the future in close relationships: condition comparisson	.00	.00	12.60	.00	.00	5.10	17130.00	−2.60	.009**	.13	200.34	185.85	N													
	Certainty (Certain)^1^	In both tasks, but in different comparisons														.00	2.70	30.50	.00	.00	17.20	7781,50	−2.11	.035	0,13	147.54	130.67	N
	Social Processes (Social)^1^	Thoughts on the future in close relationships: within narrative condition														7.69	7.95	82.60	6.67	10.91	54.80	7215.50	−2.53	.011*	.15	150.52	124.59	N
	Other references to people (Othref)^1^	Thoughts on the future in close relationships: within narrative condition														1.91	5.00	54.20	.00	4.17	36.60	7592.50	−2.07	.039*	.12	148.54	128.64	N
	Humans (Humans)^1^	Thoughts on the future in close relationships: within narrative condition														.00	4.35	46.80	.00	3.33	31.20	7571.50	−2.18	.029*	.13	148.65	128.41	N
	Total pronouns (Pronoun)^1^	Thoughts on the future in close relationships: within narrative condition														12.50	9.63	87.90	10.00	15.19	68.80	7187.50	−2.56	.011*	.15	150.67	124.28	N
	Total third person pronoun (Other)^1^	Thoughts on the future in close relationships: within narrative condition														.00	2.17	30.00	.00	.00	16.10	7667.50	−2.36	.018*	.14	148.14	129.45	N

Note: *p < .05, **p < .01, ***p < .001.

^1^Variable from the Linguistic Inquiry and Word Count (LIWC) program [48,49], ^2^ Variable from Literary Exploration Machine (LEM, [50]).

Usage percentage: Percentage of responses containing at least one word from the category.

#### Participants after the narrative framing will write more coherent descriptions of breakup reasons.

The comparison of study conditions (ANCOVA, with current suffering after breakup, frequency of counterfactual thoughts in the last week, the time of engagement in the experimental modification tasks from first stage as covariates) showed intergroup differences at the level of statistical tendency (*F*(1;402) = 3.19; *p* = .075; *eta*_*p*_^*2*^ = .008), with narrative set participants writing more coherent breakup descriptions (M = 2.95; SD = 1.14; M_adj _= 2.94; 95% CI (2.79; 3.09); N = 199) than those from the control set (M = 2.75; SD = 1.03; M_adj _= 2.75; 95% CI (2.60; 2.90); N = 208). The ANCOVA results showed that the covariates: current suffering after breakup (*p* = .657) and frequency of counterfactual thoughts in the last week (stage 1; *p* = 0.395) did not significantly affect the coherence of breakup reasons description. However, the time of engagement in the experimental modification tasks was significantly connected with it (*F*(1;402) = 7.35; *p* = .007; *eta*_*p*_^*2*^ = .018), with the longer time participant had spent on experimental modification tasks the higher was their breakup description coherence (*r*(407) =.13; *p* = .008).

The coherence of breakup reasons description differed between narrating and non-narrating participants (*z* = −4.23; *p* < .001; *r* = .24). Women who created the self-story presented their breakup reasons more coherently (N = 199; Me = 3.00; IQR (Q1–Q3) = 1.5 (2–3.5)) than those who did not provide a narrative account (N = 106; Me = 2.00; IQR (Q1–Q3) = 1.5 (1.5–3)). Supporting ANCOVA to verify if the inclusion of the perceived relationship significance (that differs between narrating and non-narrative participants) would reshape this result show that the significance of the ended relationship is not predictive to the levels of coherence of breakup reasons description (*p* = .514/.524 with additional covariant) and its inclusion does not affect the significance of group differences (*p* < .001). Next, we checked if the presence of the additional covariant being the time of engagement in the experimental modification tasks would introduce changes and found out that while it is significantly connected with the description’s coherence (*p* = .041), the group difference remains significant (*p* < 0.001) what shows that the observed effect cannot be explained solely by time spent on the activity.

#### Participants after the narrative framing will declare higher understanding of breakup reasons.

ANCOVA showed no differences (*p* = .913) between narrative (N = 203) and control (N = 213) sets, with average declared understanding of breakup reasons at 71.27 (SD = 27.17; N = 416), showing high perceived clarity regarding breakup reasons. Among three included covariates (when assessed in a model at the same time) the frequency of counterfactual thoughts in the last week (*p* = .548) and the time of engagement in the experimental modification tasks (*p* = .668) did not significantly predict declared breakup understanding. However, the current suffering after breakup did (*F*(1;411) = 40.74; *p* < .001 *eta*_*p*_^*2*^ = .09). The higher the participant’s current suffering after breakup the lower understanding of the past relationship is declared a week later (*r*(416) = −0.38; *p* < .001).

Differences in declared understanding of breakup reasons emerged in intra narrative set comparison (*z* = −3.07, *p* = .002, *r* = .17). Narrating participants had a higher level of declared understanding of the past relationship (N = 203; Me = 80.00; IQR (Q1–Q3) = 42 (56–98)) than those who did not provide a narrative account of the broken relationship (N = 117; Me = 62.00; IQR (Q1–Q3) = 51.50 (38.50–90)). Supporting ANCOVA analysis to gain insight into possibility of changes in the main effect after inclusion of the perceived relationship significance as a covariant showed, that although it predicts the declared understanding of breakup reasons (*p* < .001/ < .001 with additional covariant), the main effect of the presenting self-story or not remains significant (*p* < .001). Introducing to the model the time of engagement in the experimental modification tasks showed that it is not connected with the declared understanding (*p* = .710) and the group’s difference significance persists (*p* < .001).

#### Participants after the narrative framing will experience more thoughts about the future.

There were no differences between the conditions within the main (χ²(4, N = 401) = 5.55; *p* = .236) and detailed thematic categories of thoughts’ themes (χ²(8, N = 401) = 8.19; *p* = .415) (responses not suitable for evaluation were excluded from the analysis). However, looking into the moderating role of experienced Event Related Intrusive Ruminations (in stage 1) and solely considering interest or reluctance in future thinking, participants with high levels of intrusive rumination (frequency sum of ≥ 17) in the narrative condition (N = 86) showed a higher interest in future thinking (87.2% of their responses were clarified as future-oriented) than those in the control condition (74.7%; N = 91) (χ(1, *N* = 177) = 4.44; *p* = .035; φ = .16).

The act of narrating or non-narrating the past relationship affected the content of future thoughts in the last week (analysis of detailed categories: χ²(8, *N* = 296) = 21.52; *p* = .006; *V* = .27; 27.8% of cells with a predicted number less than five) (responses not suitable for evaluation were excluded from the analysis). Among those who created a self-story (N = 201), 3.5% (standardized rest −1.9) stated no thoughts about their future in close relationships (N = 7), which was about four times less than in those who failed to write a narrative – 14.7% (N = 14 from 95; standardized rest 2.8).

The character of the used analysis made controlling for differences in the first stage not feasible.

#### Higher plot structuring of self-story will be connected with more beneficial results.

The next analyses included only narrating participants (N = 204), that is, those from the narrative condition who created a self-story.

**Correlations:** The r-Pearson correlations of plot structuring showed that in prestest, it was weakly connected with perceived relationship significance (*r*(204) =.15; *p* = .028) and the Event Related Deliberate Ruminations (*r*(204) =.20; *p* = .004). Among the cognitive variables from Stage 2, the only connection was to deliberate ruminations (*r*(203) =.14; *p* = .043). Additionally, there was a correlation with coherence of the description of breakup reasons (*r*(202) =.24; *p* < .001). Correlations with linguistic variables were calculated only with those present in more than 20% of the responses. Only those meeting the requirement of a linear relationship, not resulting from outliers and providing worthwhile insights, are reported.

Correlation statistics are included in Table 6.

**Table 6:** Statistics of r-Pearson correlations between narrative structuring and chosen language variables in both open tasks.

	The breakup reasons description (N = 191)			Thoughts on the future in close relationships (N = 195)		
Variable	*r*	*p*	Usage percentage	*r*	*p*	Usage percentage
Word Count (WC)^1^	.32	<.001***	100.00	.35	<.001***	100.00
Verb^2^	.28	<.001***	96.90	.30	<.001***	98.50
Past tense verb^2^	.26	<.001***	95.30			
Future tense verb^2^				.15	.038*	66.70
Present tense verb^2^	.21	.003**	54.50	.20	.005**	71.30
1st person singular verb^2^	.22	.002**	63.90	.20	.005**	93.30
3rd person singular verb^2^	.25	.001**	84.80	.24	.001**	59.50
Infinitive verb^2^	.15	.044*	59.20	.20	.006**	59.50

Note: *p < .05, **p < .01, ***p < .001.

^1^Variable from the Linguistic Inquiry and Word Count (LIWC) program [48,49], ^2^ Variable from Literary Exploration Machine (LEM, [50]).

Usage percentage: Percentage of responses containing at least one word from the category.

In both tasks, there were positive correlations with word count (LIWC), number of verbs, present tense verbs, 1st person singular and 3rd person singular verbs, and infinitive verbs (LEM). Distinctive correlations with past tense verbs and future tense verbs (LEM) were observed in past-oriented descriptions of breakup reasons and future thoughts.

**Subgroup comparisons:** Next, we compared recoded subgroups of those creating stories of incomplete (narrative structuring score 1, N = 63), basic (score 1.5–2, N = 69), good (score 2.5–3, N = 46), and excellent narrative structure (score 3.5–4, N = 26).

Differences between the subgroups emerged in the coherence of breakup reasons description: *H*(3) = 15.67; p = .001; ∊^2^ = .08; *N* = 199. Individuals who composed self-stories with incomplete narrative structures later constructed less coherent descriptions of breakup reasons (N = 61; Me = 2.00; IQR (Q1–Q3) = 1.00(2–3)) compared to those whose self-stories possessed higher levels of narrative structure (basic: *p* = .022, N = 68; Me = 3.00; IQR (Q1–Q3) = 1.87(2,13–4)) (good: *p* = .007, N = 45; Me = 3.50; IQR (Q1–Q3) = 2.00(2–4)) (excellent: *p* = .014, N = 25; Me = 3.00; IQR (Q1–Q3) = 1.25(3–4.25)).

There were also some differences in language use between the subgroups. Participants who wrote self-narratives with incomplete structure used fewer words (WC – LIWC) in breakup reason descriptions (Me = 20.00; IQR (Q1–Q3) = 22.00(12–34); N = 61) than those with basic (Me = 30.50; IQR (Q1–Q3) = 32.25(20.25–52.50); N = 68; *p* = .031), good (Me = 47.00; IQR (Q1–Q3) = 47(24.50–71.50); N = 41; *p* < .001), and excellent narrative structures (Me = 56.00; IQR (Q1–Q3) = 61(20–81); N = 21; *p* = .004) (*H*(3) = 21.03; p < .001; ∊^2^ = .11; N = 191). The same pattern (*H*(3) = 20.43; p < .001; ∊^2^ = .11; N = 195) was observed in the length (WC – LIWC) of thoughts on the future in close relationships with participants with incomplete story structure giving shorter responses (Me = 17.00; IQR (Q1–Q3) = 13(12–25); N = 60) than the other subgroups (basic: Me = 24.00; IQR (Q1–Q3) = 20.5(16–36.5); N = 69; *p* = .033, good: Me = 27.00; IQR (Q1–Q3) = 34.25(18.50–52.75); N = 44; *p* < .001, excellent: Me = 31.00; IQR (Q1–Q3) = 40.50(15.75–56.25); N = 22; *p* = .014).

Regarding verb use (LEM), in the description of breakup reasons (*H*(3) = 17.53; p < .001; ∊^2^ = .09; *N* = 191) and thoughts on the future in close relationships (*H*(3) = 18.26; p < .001; ∊^2^ = .09; *N* = 195), participants who wrote a self-story with an incomplete story structure used fewer verbs than those with good and excellent story structures. Breakup reasons statistics: Incomplete: Me = 4.00; IQR (Q1–Q3) = 5.00(2–7); N = 61. Basic: Me = 6.00; IQR (Q1–Q3) = 8.50 (3.25–11.75); N = 68. Good: Me = 8.00; IQR (Q1–Q3) = 9.5(5–14.50); N = 41; *p* < .001, Excellent: Me = 11.00; IQR (Q1–Q3) = 10.50(3.5–14); N = 21; *p* = .029. Future thought statistics: Incomplete: Me = 3.50; IQR (Q1–Q3) = 3.00(2–5); N = 60. Basic: Me = 5.00; IQR (Q1–Q3) = 5.00(3–8); N = 69. Good: Me = 6.00; IQR (Q1–Q3) = 7.00(4–11); N = 44; *p* < .001, Excellent: Me = 6.50; IQR (Q1–Q3) = 7.25(3.75–11); N = 22; *p* = .020.

More past verbs (LEM) in breakup reasons descriptions (*H*(3) = 13.64; p = .003; ∊^2^ = .07; *N* = 191) were present in the responses of those who wrote good (Me = 5.00; IQR (Q1–Q3) = 4.00(3–7); N = 41; *p* = .008) and excellently structured narratives (Me = 6.00; IQR (Q1–Q3) = 7.5(2–9.5); N = 21; *p* = .038) than responses of those whose self-stories had incomplete narrative structure (*Me* = 3.00; IQR (Q1–Q3) = 2.00(2–4); *N* = 61). There were no differences between any condition and those writing basic stories (*Me* = 3.00; IQR (Q1–Q3) = 5.00(3–7); *N* = 68). There were no differences in the number of future tense verbs (LEM) used in thoughts on the future in close relationships (*p* = .077) or causation word use (LIWC) in any task (breakup reasons description: *p* = .465; future-oriented thoughts: *p* = .112).

### Analysis of potential factors preventing narrative structure classification

The first article author undertook a qualitative analysis on the responses that trained coders classified as non-narrative. The findings revealed that about one-third of them (31.4%) did not provide any valuable information about the ended relationships. 20.3% of the responses provided some insight into relationship or breakup but without a clear coherent structure, be it narrative or argumentative. 22% of participants justified their breakup, primarily by sharing their opinions, reflections, or arguments for the relationship end. 10.2% presented a chronological chain of events that, unfortunately, lacked a narrative thread and could be seen as a simple report of the events from the time of the relationship. 14.4% were close to providing narrative self-stories but failed at describing the whole relationship, maintaining a narrative thread throughout the description of its different parts, and/or describing explicitly how the breakup occurred. Lastly, 1.7% provided narrative/narrative-like responses, but because they were focused on different topics, the relationship breakup was a subplot of their story and was not sufficiently described. Example responses fitting each category are presented in Table 7.

**Table 7:** Categories of participants’ responses of non-narrative structure.

	N	Percentage	Example answer
Failure to answer the question	37	31.4	Leaving response empty, submitting nonsensical words or interpunction marks; avoiding answering the question – declaring outright refusal to answer or not remembering the history of the relationship; given response does not provide any knowledge about the relationship – answer truncated, possible technical error in completing the survey^1^
Conveying a small amount of information about the relationship in a non-narrative and non-argumentative manner	24	20.3	1. I sacrificed everything for this boy because I went abroad but it taught me a lot that I can handle myself in any situation 2. It was my second relationship, we were happy but unfortunately not always two people despite wanting can be together 3. We separated because of the infidelity of the partner.
Justification for the breakup of the relationship - opinion/reflection/argumentation	26	22.0	1. We separated due to the neglect of our relationship. One side tried very hard, and the other side did not try at all. Of course, everything came with time. We were not able to talk to each other afterwards, and when it came to talking it would come out as an argument, because one would jump on the other. In the end, my desire for it to be good came to an end, because in most situations I was the one who reached out, I finally said enough and ended the relationship. 2. We met at work and were together for a month. However, it was noticeable the lack of interest on the other side and little desire to solicit contact hence the decision to end the relationship.
Chronological presentation of events, with an absence of the narrative thread	12	10.2	1. We met on the Internet when we were 14 years old, after 2 years we started dating, everything was going well, we were planning a baby in 2/3 years, but at some point everything started to break down, we decided together to separate, we live in harmony. 2. We met at his prom. He was the first to confess his feelings. Our relationship survived a month-long separation caused by my work abroad. After a few months of relationship we moved in together. We separated after almost 3 years due to my health problems.
Partial narrative	17	14.4	We were both trying for a baby we were very much in love with each other until I got pregnant. Then things started to change, for the worse. He started to be at home less often explained by work, mom, brother. Arguments began. Then summer came and he suddenly has a hobby of fishing he left me at home every weekend even though I cried and asked him to stay home with me but he didn’t give a damn about me. At the end of the pregnancy he stopped bringing money home to live on and still resented because he had to buy a car for himself. He was not interested in bills, doctor, examinations, medicines, milk, pampers...nothing. finally 2 weeks after the birth I told him to move out of my house even though I loved him I left him, because the child for me is the most important thing and he is not suitable either as a father or as a partner for life. And that’s the end of the story.^2^
Narrative not on topic	2	1.7	I have always had a friend with whom I was in love, and he with me (we have known each other for 10 years). He wanted to be with me, and somehow I was afraid to start a relationship with him, because I was afraid that our relationship would break down. Eventually he found a girlfriend, and after some time I also found a boyfriend. Nevertheless, I did not lose contact with my friend, because we commute together. One time I was talking to my friend about my boyfriend. He told me words to the effect that he knows that I don’t love him. These words touched me and I began to wonder what he actually felt. Eventually the next day I broke up with this boyfriend. However, it was a parting in harmony. Now 8 months have passed since that breakup, and my friend still has a girlfriend. Every now and then I think about this breakup, however, more, I regret the fact that with my friend it came out as it did.

1 Empty responses and those containing nonsensical words or interpunction marks were not scored by trained coders due to their explicit non-narrative character

2 The story describes only episodes of a relationship falling apart and the breakup. There is no information about meeting the partner, the decision to be together, or more elaboration on briefly mentioned happy times.

## Discussion

### Findings and interpretation

In line with narrative theory [5,9,13,18,52,94] and the results of previous experimental studies [20,21,29,34,42], we expected that activation of the self-story framing of a broken love would positively influence thinking quality on the past and future in their own close relationships. Narrative framing should enhance the understanding of the causal links in the development and break-up of the past relationship and in imagining and planning possible relationships in the future. More importantly, we expected that the level of structuring the narrative plot would enhance these effects. They should be revealed in the description of the reasons for the breakup, declared understanding of breakup reasons, and thoughts on possible close relationships. We took into account the readiness and ability of women to engage in narrative thinking. In addition to a direct comparison between the experimental and control conditions, the analyses included two characteristics of participants in the narrative set: (1) whether they provided self-story or not and (2) the narrative quality of the provided description: to what degree does a broken love description embody the narrative structure? We assumed that asking a person to narrate, and their willingness and ability to narrate, created an experimental narrative set.

The experiment with the narrative and control sets confirmed the hypothesis that activated narrative thinking – especially when combined with the willingness and ability to narrate one’s own broken love – results in a better understanding and imagining of the causal structure of the past relationship and expected or possible new ones. Text analyses using LIWC (Linguistic Inquiry and Word Count [48,49]) and LEM (Literary Exploration Machine [50]) indicated that women with higher activation of a self-story (narrative condition and narrating participants from the narrative set) produced longer, more causally connected reflections that were more action-oriented and temporally appropriate than open responses from their counterparts. These results confirm the hypothesized cognitive processes induced by self-story activation, reflected in language usage. Elaborated responses employing more verbs of matching tense (e.g., past tense for breakup reasons and future tense for thoughts on future relationships) may indicate improved mental simulation, aligning with findings on the connection between narrative mindset and inference generation [34] and the inclusion of personal perspective in problem-solving advice [42]. Causation words, including “because,” “effect,” and “hence,” serve to establish explanatory links. According to Boals and Klein [107], these terms are more prevalent in segments of narratives of broken love that describe breakup and its aftermath than in those depicting the earlier stages of the relationship. This disparity can be attributed to individuals’ greater need to organize their thoughts in the face of the most challenging situations. Pennebaker et al. [108] demonstrated that an increase in the utilization of causation words and insight words, such as “think,” “know,” and “consider,” in trauma disclosures is predictive of improved physical health. An elevated number of causation words in the reflections of participants with activated self-stories indicates improvement in the creation of causal connections [33,34,51] which could be helpful in the cognitive processing of one’s situation [108,109]. Verbs underpin narrative thought by representing actions [57]. It seems worthwhile to address in future analyses verb content used by participants after self-story activation. First, to verify whether they are action verbs characteristic of narrative thinking (or state verbs typical of impression formation) [51]. Second, to look into the causality of actions that verbs might attribute [110,111] leading to insights into agency distribution between actors and situational factors.

The narrative framework was expected to increase the coherence of the presentation of breakup reasons. This hypothesis was supported by an intra-narrative group comparison, in which individuals who shared a breakup self-story (compared to those who did not) provided a more coherent breakup reasons description seven days later. A comparison of the narrative and control sets revealed a similar trend, although it reached only a statistical tendency. These findings concur with language analyses showing more elaboration in breakup explanations and stronger linguistic causal associations in the responses of participants with activated narrative thinking. People often face the task of identifying believable reasons for a breakup, both for themselves and others [77,112]. Hartmann and Trpin [113] suggest that coherence between premises and conclusion can strengthen an argument. Presenting the breakup reasons in a coherent manner may lead others to perceive the relationship’s end as more justified, resulting in the person presenting the argument being viewed as more trustworthy. This can be highly beneficial in fostering close relationships.

Consequently, narrative framing also increased the declared understanding of breakup reasons. This was confirmed in a comparison within the narrative set: participants who described a self-story of broken love a week later declared a higher understanding of the breakup than those who did not provide a narrative account, as required. This suggests that narrative framing improves breakup understanding, but only for those who are able and willing to narrate. However, no significant differences were observed between narrative and control conditions.

Narrative framing effects were outlined more for reflections concerning the personal past than for those on the personal future. In future-oriented thoughts, we observed only a couple of effects: being of a general character or of a small range. In the condition comparison, no differences were observed in the content of thoughts on the future at the thematic category level. However, differences emerged among participants who were high in intrusive rumination. In this subgroup, those from the narrative group showed a higher interest in future thinking (i.e., more experienced future-oriented thoughts) than their control counterparts. This effect highlights the potential of narrative thinking to shield from the consequences of other present challenging cognitive processes [29]. In an intra-narrative group comparison, women who refrained from sharing their self-story (non-narrating) were four times more likely to express no thoughts on the future in close relationships in the last week (14.7%) than women who shared their self-story (3.5%). Although retrospection and prospection are constructive processes, they differ in their nature. Reflecting on the past involves considering facts and events realistically. By contrast, future thoughts focus more on personal goals, wishes, or key situational factors, with less emphasis on veridicality [114]. Past reflections are more closely connected with contents activated through narrative framing, as concrete experiences and their details are prepared as building blocks for construed narrative [19,40,115]. Nonetheless, noting the impact of narrative thinking on future-oriented thoughts expands our understanding of its influence and shows that processes observed in laboratory studies on narrative mindset can predict real-life consequences of narrative thinking [34].

The final exploration area was the association between the narrative quality of a construed self-story (embodying the canonical story structure – plot structuring) and observed effects, with a positive connection between its level and beneficial functioning. The relationship between plot structuring and the perceived relationship significance as well as Event Related Deliberate Ruminations (both before and after experimental modification), was found to be positive. Additionally, there was a correlation between coherence of breakup reasons description and plot structuring, with higher levels of plot structuring co-occurring with more coherent breakup reasons presentation. Higher plot structuring was linked to more elaborated reflections that contained more verbs, including those indicating a suitable time orientation (a higher number of past tense verbs used to describe breakup reasons and more future tense verbs used to think about the future in close relationships). The study did not reveal any correlation between plot structuring and the creation of causal connections in reflections created a week after self-story activation, leaving this aspect of the hypothesis inconclusive. Subsequently, we examined the differences in the cognitive processes of women characterized by varying the narrative quality of construed accounts of the past romantic relationship. Women who wrote self-stories of at least a basic story structure created more coherent breakup reasons descriptions than participants with an incomplete narrative structure of the broken love description. Participants who created self-stories with incomplete narrative structures wrote less elaborate reflections than did those with higher levels of self-story plot structuring. For verb use, a higher number was observed in the reflections of participants writing self-stories with good or excellent story structures than in responses of those with incomplete structures. The same pattern emerged for the presence of past-tense verbs in the description of breakup reasons. No differences were found in the use of causation words or the number of future verbs in thoughts on the future. Improved causal binding of reflections was only observed in group comparisons (activation or lack of activation of narrative thinking), and was not differentiated by the level of narrative structuring of a self-story. This suggests that psychological benefits can be reaped, even in the case of suboptimal narrative thinking, as observed in the creation of an incomplete story structure. The conveyed narrative content may be affected by numerous factors, ranging from motivation or situation to the willingness for self-disclosure [116]. Nevertheless, the quality of the written text is of second importance to the cognitive processes in the participant’s mind, where narrative thinking might be activated, leading to reflection on and elaboration of inner narrative in a participant’s daily life outside of participation in a study [11,18].

The results supporting the hypotheses were present in both chosen comparisons: between the experimental groups (narrative vs. control) and within the narrative set (narrating vs. non-narrating participants). However, the second approach, which involved comparing women who shared their self-stories of broken love with those who did not despite being asked to do so, yielded a more significant confirmation of the hypotheses. A comparison within the narrative group provides the most straightforward contrast for engagement or disengagement in narrative thinking. The presence of some of the effects in both comparisons (processes observable in language use – elaboration, causal binding, action, and time–orientation) confirms that they are the consequence of self-story activation. It is feasible that certain control group participants engaged in narrative thinking spontaneously, prompted by reflecting on the assigned nonnarrative study’s contents [51,117], and subsequently experienced benefits similar to those who underwent experimentally induced narrative framing. In case of naturalistic experiments there is no way to prevent the possibility of the narrative thinking in a control group’s participants. Therefore, the experimental conditions should be seen as group embodying different levels of self-story activation: high in narrative group and low (but not completely absent) in the control condition.

To ensure, to the best of our ability, lack of or low level of self-story activation, we observed participants’ responses to the pilot studies of our materials. Through that, we saw that when there is unrestricted space to provide the open answer, the risk of it turning into narrative-like content is always present. To tackle it, instructions to the open tasks of the control group highlighted that the response needs to be short (e.g., asked to answer using keywords, to write answers no longer than one sentence, to create lists), asked for recalling memories connected by topic and not chronology, provided examples of responses of a non-narrative character (such as a list of traits), and gave visual clues for providing short responses by incorporating small boxes for submitting them. Despite the evident disadvantage of undertaken approach to defining research conditions, it is necessary to ensure compliance with the principle of the only difference. Projects that use a task of writing on another topic as a comparison to self-disclosure [e.g., 118,119] are limited in their ability to demonstrate the benefits of a particular response structuring. This is because their control condition lacks the cognitive activity of evoking similar episodic memories from the important event that are at the center of the guided intervention of narrative structuring [31].

Within the narrative condition, the reasons for a narrative structuring failure or the decision to not share one’s self-story require further exploration. They were not the result of worsened psychological functioning as assessed by the Event Related Ruminations, frequency of counterfactual thoughts, or current suffering after breakup. Qualitative analysis of the responses that trained coders classified as non-narrative done by the first authors reveals that about one-third (31.4%) of non-narrative participants could be seen as unwilling to share broken love self-story. The rest of the group provided some information about the relationship or its breakup, through which they showed their engagement in research activity. One-fifth struggled to present their experiences coherently, and a similar number decided to share them in the form of an argumentation, not a story. Close to one out of ten non-narrating participants failed to connect experiences by the narrative thread, resorting to the presentation of a chronological chain of events. Texts closest to the self-stories of broken love either failed to present the whole length of the relationship narratively (14.4%) or were mainly focused on an entirely different topic (1.7%). It suggests that for most participants, either a lack of trust in the research procedure or problems in forming coherent narratives lead to submitting non-narrative answers. The post hoc character of the analysis and its results being based on first author classifications and not trained coders’ statements declared at the moment of the decision process, ask for approaching its results with utmost caution. The mechanisms responsible for the narrating failure have been proven to be fair from simple to pinpoint and require further explorations [59,62].

The study’s results indicate positive connections between the self-story’s narrative quality (plot structuring) and observed thinking effectiveness. These findings are particularly significant as they support the hypothesized mechanisms of the narrative framing influence [20,21], aligning with the prevailing scientific consensus on the favorable association between the coherence of self-stories and psychological functioning [54].

The current study adopted a short horizon of one week for delayed effects measurement. Frattaroli’s meta-analysis [22] of experimental disclosures indicates stronger overall and psychological health effect sizes for studies measuring effects less than a month after the intervention compared to longer periods. Despite this, most studies in the meta-analysis chose extended measurement periods (average three months). Studies closest to this project measuring self-story activation impact also used long measurement times (2 weeks to 7.5 months) [20,21,29,30]. The emergence of some effects only after longer periods (2 weeks vs. 3 months) provides rationale for planning long measurement delays [20]. However, this complicates the mechanism’s description behind the observed effect. For instance, while we know the starting point is activating a self-story, explaining its translation into favorable cardiac performance remains speculative [30]. The short-delayed period in this study allows connecting effects to narrative thinking and identifying likely mechanisms. Effects such as increased activated memories of the ended relationship, thoughts about future life in close relationships, and deepened ability to construct cause-effect relationships can fade quickly, forming possibly the basis for more complex consequences of narrative thinking (e.g., increased sense of meaning in life [20]). Future research should focus on measuring near-term consequences of narrative thinking through intensive daily measurements for a month, allowing connection with later effects. This would capture the dynamic consequences of narrative thinking. Most likely, as long as a person retains memories of a former relationship [41], consequences associated with narrative structuring may persist and develop, evoked by incoming experiences [120].

### Limitations

This study had several limitations that warrant further consideration. The observed effects were relatively modest in size, which raises the possibility that some effects of narrative framing may have gone undetected due to the limited sample size. The exclusion rates of 36.65% for the narrative group and 29.45% for the control group on verification of participation in the experimental tasks may seem high. However, they are consistent with a previous study by Boals and colleagues [43], who found that 50% of participants provided answers that were too brief for scale-based evaluation of insight into breakups. Future studies on narrative framing effects should account for the weak effects and high percentage of participants failing to complete the experimental tasks in their group size estimation. Current project unfortunately has not included any activities (as writing on non-relationship related topic) that would grant insight if non-narrative responses are a result of inability to engage in narrative thinking or rather lack of trust in the study procedure or a reluctance to share personal information. It would be worth addressing in future studies.

The selection of participants for the project was restricted to obtain a homogenous sample. Emerging adult women after a heterosexual breakup were chosen due to the high likelihood of successful recruitment and possible significant impact on their future life trajectory. Obtained results can be only connected with this gender and relationship situation. Romantic relationship studies tend to gather primarily female-predominant samples (47–100% females in the meta-analysis of [23]. Similar trend is found for studies on experimental disclosures (averaged 34% of men in studies included in Frattaroli’s metaanalysis [22]. Self-story activation studies most important for this project fail to assess gender differences because of lack of man in a study sample [20,21] or declaring their insufficient number ([31]; studies not clarifying reasons for lack of comparison but being based on the connected dataset – [29,30]). To achieve more diverse and inclusive representation, future projects should consider incorporating participants from various gender and relationship backgrounds. Metanalysis of experimental disclosures [22] suggest lack of differences between man and women in the strength the intervention’s effects, similarly none were found in a study of expressive writing on dealing with a breakup [119]. Therefore, it is most likely that men may significantly benefit from using self-story intervention by uncovering distress in a secure environment, gaining from enhanced thinking effectiveness, and potentially becoming more open to seeking professional help [121,122]. Current study refrained from inviting homosexual women after breakup, recognizing that for them building a self-story on broken love might be more challenging due to fewer societally shared stories on dealing with this situation [18] and possible met unsupportive social reactions while sharing it [86,87]. The positive correlation between listening to intergenerational stories within the LGBTQ+ community and psychosocial identity [123] suggests a general advantage of narrative usage, indicating that there should be no ethical concerns preventing the exploration of the impact of the narrative framing of one’s breakup from non-heteronormative relationships.

### Project’s practical relevance

Dealing with a romantic relationship breakup, which in the past might have been mostly a matter of a particular developmental period [68], evolves into a life-long needed competency. It is a consequence of a cultural shift from relationships being societally guided constructs to being a part of a strictly private domain [64]. There, its significance holds firm [70] while tolerance for dissatisfaction and harmful elements of relational life (e.g., infidelity, violence) decreases [64,67,89]. However challenging and stressful, breakups are less damaging to psychological functioning for emerging adults than maintaining unsatisfactory bonds [23]. Since emerging adults strive to build personal resiliency to function alongside and deal effectively with stressors [75], learning how to understand oneself, partner, and love relationships to realize one’s goals is crucial. Narrative thinking seems to be a promising tool for this task because it concentrates on actors and their guiding motives [9,13,124]. An ideal post-breakup intervention should address dealing with the past and support a person in embracing thoughts and activities that foster a fulfilling future. Previous studies have found that understanding the reasons for breakup is connected with better well-being, a higher likelihood of entering a new relationship, and higher romantic competence [43,78]. Narrative framing effects in this area, combined with its influence on thinking about the future, make it a promising well-being intervention for individuals recovering from a romantic relationship breakup. This approach is particularly appealing, given the current lack of scientifically verified interventions for those experiencing such difficulties [25,27,28].

This study showed not everyone is able or willing to engage in creating a self-story, thus this intervention will not be universally applicable. Still, if a request to create a self-story would proceed a meeting with therapist, even or maybe especially when it would end in failure of creating a comprehensible story it could gain valuable insight into participants psychological functioning [125]. Writing a self-story, similarly to expressive-writing (sharing one’s thoughts and feelings without any structural requirements) could be advised for anyone willing to reflect on a personal experience, with a clear warning that it’s effects are not guaranteed and while one might wish to feel better after engaging in it, the effect might be contrary due to evoked unpleasant thoughts [126]. Advantage of engaging in self-story writing compared to expressive writing lies in the likelihood of more beneficial effects (found in area of divorce – [29,30]) and possibility of sharing one’s story with others to elicit social support. Even well-crafted stories are worth consulting with trusted others or specialists since they might reaffirm harmful beliefs [81]. As a psychoeducation or support tool for those failing to frame personal experiences narratively, trainings focused on writing self-stories could be introduced [127,128]. Especially teaching adolescents and emerging adults [24,70] what beliefs from culturally shared love stories are beneficial for relationship functioning and which are not [18,128,129] could facilitate successful breakup management and shape their developing life stories [130–132]. In the next step, it could increase awareness that life can be seen narratively [133,134], encouraging using stories as coping and motivational resources.

In life studies on self-story influence, we unavoidably deal with various breakup situations, making pinpointing the consequences of particular story structuring and incorporated contents challenging [107,109]. Laboratory studies on narrative mindset should focus on understanding how causal links between events are formed, how the impact of an element changes based on its position in the narrative, and which type of event processing produces the desired results.

The results of our study must be viewed from a broader cultural perspective. This will allow us to show the social and cultural conditions that are necessary—or conducive—to the ways of experiencing a breakup from a romantic relationship identified in this study. Our study was conducted in contemporary Poland, a country that culturally belongs to the circle of Western Europe and North America. Contemporary individualistic cultures, prevailing in these countries, place a higher value on individual needs and romantic love, reflecting a shift from the traditional views of love. In romantic love, passion is central. Such relationships—their stories, the problems encountered, and the breakup or happy ending—are presented in literature, mass media, and Internet communications. The difficulties and possible end of a woman’s romantic relationship are her problems, not those of her family and immediate environment. The emotional component of the breakup is to be highlighted, as it matches the cultural focus on individual needs, well-being, and the modelled ways of experiencing this situation. Our study focused on romantic relationships in this cultural area, and its results indicate that the narrative framing of broken love can lead to a number of beneficial consequences. Personal emotions and motives are the building blocks of self-stories. Such self-stories, when activated, may positively or negatively influence our functioning.

Cultural anthropology and sociology indicate that in many societies, a different way of understanding and evaluating romantic relationships between men and women predominates. In traditionalist cultures, primarily emphasized is the social significance of the romantic relationship and its procreative role. This shapes the way love is understood and experienced by the women and men involved. A romantic relationship and its subsequent phases result from the well-defined social roles of the partners and from the culturally defined finalization of love – the formation of a new family and procreation. This does not mean that there is no room for feelings; the point is that the content of these feelings is shaped more by internalized cultural patterns and the expectations of the family or others than by emotions, relatively independent of the social norms and expectations.

This is illustrated for example in recent data on the differences in experiencing love and couple happiness among Spanish couples and Moroccan couples living in Southern Spain [135]. It appeared that in more collectivistic cultures, like Moroccan, family and religion heavily influence relationship decisions, potentially leading to a more stable relationship focused on long-term commitment and mutual support. The valued love is less passionate but more companionate, intimate, and practical. Spanish women are engaged in less stable relationships, more influenced by several agents, and value romantic love as most important in the relationship, also value intimacy more than commitment and passion. Functioning in a collectivistic culture can lead to a certain narrowing of the content and role of experienced emotions. Thus, the regulative role of narrative framing of broken love might be different than that observed in the current study in an individualistic culture. In a cross-cultural study, Śmieja et al. [136] found that higher self-direction at the country level increased the well-being disparity between single and coupled individuals, while higher conformity reduced this disparity through its connection to meaning in life. Collectivism affects developmental patterns among emerging adults, with higher collectivism linked to socio-emotional profiles and lower levels to self-efficacious or high/balanced profiles [137]. Although the use of narratives is universal [5,7], culture may shape one’s views on close relationships and developmental trajectories, which may subsequently affect the consequences of narrative structuring of broken love, most likely through emotional and motivational factors. Future projects should verify the role of culture as a moderator in this type of personal experiences. An additional factor worth considering in such studies is the social character of the construed narratives. Private self-stories versus those created to help others (e.g., to warn against partner choice mistakes) could lead to different psychological results across countries with varying collectivism levels.

## Conclusions

The current study has demonstrated the beneficial effects of activating the self-story of broken love on thinking effectively in areas of the personal past and potential future in close relationships. These effects support theses of the narrative theory [5,9,13,18,52,94] and show that results of laboratory studies on narrative mindset are predictive for the real-life [34,42]. The findings of this study are of significant importance and reaffirm the value of self-story structuring (narrative coherence) as an important factor connected with well-being [20,21,54,56]. For further implementation into studies on narrative thinking effects in the real life of participants (self-story activation), based on current project results, we postulate to:

1. Measure its close-range effects (use short delay periods) that can be straightforwardly connected with basic research.
2. Incorporate open responses that allow observing generative and constructive properties of narrative thinking.
3. Use various methods of effects measurement, most importantly trained coders and linguistic analyses.
4. In planning contrast to the experimental narrative group, include active control tasks that would also evoke recollections connected with challenging experiences.
5. As a secondary comparison group, examine participants who fail to provide narrative responses despite being asked to do so.

The presented project extends our knowledge of the beneficial consequences of self-story writing intervention [20,21,29,30], observing it in a new area of breakup from informal romantic heterosexual relationship experienced by emerging adult women. Through that, it hopes to encourage further studies on self-story activation, particularly in other challenging interpersonal experiences. Importantly, it underscores the need for further studies on the consequences of narrative thinking to provide support for evidence-based narrative therapeutic interventions.

## Supporting information

S1 Appendix Participation quality Control Set – Answers Completion. (PDF)

S2 Appendix Participation quality Narrative Set – Narrative Structure Assessment. (PDF)

S3 Appendix The Plot Structuring Scale. (PDF)

S4 Appendix Coherence of the Description of Breakup Reasons. (PDF)

S5 Appendix The theme of thoughts on the future in close relationships. (PDF)
