# Supplementary material for: The effects of narrative framing of own broken love on understanding the past and imagining the future in close relationships
Source: PLoS One. 2025 Nov 25;20(11):e0334973. doi: 10.1371/journal.pone.0334973 (PMC12646452; doi:10.1371/journal.pone.0334973)
Supplement: S1 Appendix — (PDF) [file pone.0334973.s001.pdf]

Control condition – answers completion

The evaluation is based on the value of the answer for the posed questions. The final indicator of quality is the percentage of complete answers.

A valuable answer is considered to be one that is a plausible answer to the question asked or that cannot be ruled out as to be seen as such by the participant (e.g., use of metaphors, self-created words, etc.). Content that does not meet this criterion is not considered a valuable answer.

In particular, avoiding answers by typing "I don't know," "I don't remember," "none," ..., -, "the same" is not considered a valuable answer (if it can be reasonably assumed that the participant should have access to the information that answers the question). Nor is it a valuable answer to repeat the same answer within the subsections of one question. In this case, only the first entry of a given answer should be considered valuable. In complex situations, the train coder decides to leave as many fields filled in as would correspond to the real value of the information provided by the participant (e.g., a participant describes two differences between herself and her partner in 10 fields, which corresponds to the values of a standard fill in two fields).

Refusal to answer is not considered a valuable answer (including its shorter form, e.g., "no" or all other manifestations indicating unwillingness to answer). This rule does not apply to the question on issues of health problems. There the refusal to answer is exceptionally considered a valuable answer.

The way trained coders work:

1. Reading all of a given participant's responses to research questions

2 Deleting all answers that the trained coder classifies as not valuable. This will enable the automatic calculation of the number of unmet tasks.

Question instructions for the control group are included in the evaluation sheet provided to the trained coders

Control procedure

Example responses from a chosen group of participants who took part in the study, though not necessarily meeting all its criteria

Experiences of a former relationship

We are interested in learning about your individual experiences, thoughts and assessments related to the relationship that ended in a breakup.

It is up to you to decide what you will share with us and how you will approach completing open-ended tasks.

Please answer freely without caring about the style or worrying about mistakes. It is as if you were doing it just for yourself. The most important thing is to keep your experiences and

thoughts real and honest. For your own sense of comfort, you may choose not to provide details or change them to something else.

Myself and former partner

Please list your interests (e.g., singing, cooking, bicycling) in a slogan.

Example response: Photography, cooking, music

Please list your former partner's interests (e.g., singing, cooking, bicycling) in a slogan.

Example response: Jogging, gym, sport

Please list the main characteristics of your personality.

You can do this by providing a list of adjectives you would use to describe yourself (e.g., open-minded, warm-hearted, hard-working...) or by listing traits (open-minded, warm-hearted, hard-working...).

Example response: Patient, empathetic, open, sincere, caring, affectionate

Please list the main personality traits of your former partner.

You can do this by providing a list of adjectives you would use to describe your former partner (e.g., open-minded, warm-hearted, hard-working...) or by listing traits (open-minded, warm-hearted, hard-working...).

Example response: Caring, hardworking, sincere, sensitive, understanding

Please list five similarities between you and your former partner.

These may include habits, traits, preferences, likes and dislikes. If possible, please briefly describe each similarity (in a few words, one sentence at the most).

	Similarities
	Me and Partner
1.	
2.	

3.	
4.	
5.	

Example response:

	Similarities
	Me and Partner
1.	Listening to the same music
2.	Going for walks
3.	Healthy eating
4.	Helping others
5.	Taking care of animals

Please list five differences between you and your former parter.

These may include habits, traits, preferences, likes and dislikes. If possible, please briefly describe each difference (in a few words, one sentence at most).

	Differences	
	Me	Partner
1.		
2.		
3.		
4.		
5.		

Example response:

	Differences	
	Me	Partner
1.	Shy	Bold
2.	Introvert	Extrovert

3.	Focused on school	Focused on extracurricular activities
4.	Reluctant to express opinions	Expresses opinions very often
5.	Not very assertive	Assertive

What made your former partner different from other men you knew? Please briefly (keyword) list these differences. They can refer to what your partner was better at, worse at, or simply different from the men you knew.

Difference 1, Difference 2, Difference 3, Difference 4 (after each "difference" there was a field to enter the response)

Example response:

Difference 1

Walking one's own path

Difference 2

Liked romantic comedies

Difference 3

Aversion to alcohol

Difference 4

Aversion to cigarettes

What was your health condition during the relationship? Did you suffer from any long-term health problems?

If so, please list them briefly:

Example response: No

What was your former partner's health condition during the relationship? Did he or she suffer from any long-term health problems?

If so, please list them briefly:

Example response: No

During the former relationship

Please list in a slogan the important life activities you were engaged in at the same time you were in a relationship.

e.g., studies, work, volunteer activities, meeting with friends, etc.

Example response: Cosmetology studies, work as a hairdresser, volunteering in a mental health foundation

Please list in a slogan the life activities that your former partner was engaged in, at the same time you were in a relationship.

e.g., studies, work, volunteer activities, meeting with friends, etc.

Example response: Mother's illness, work in a car repair shop

Where did you meet your former partner? Please indicate where this happened.

Please give a short name: e.g., university, a house party at a friend's house, a resort town.

Example response: Internet

Please list five places where you most often spent time with your ex-partner during your relationship.

This involves a general representation of the place (e.g., my apartment, university, cafe).

Places				
1.	2.	3.	4.	5.

Example response:

Places				
1.	2.	3.	4.	5.
His apartment	Cafe	My apartment	Park	Restaurants

Please sloganize the five most common topics of conversation with your former partner while the relationship lasted.

Topics				
1.	2.	3.	4.	5.

Example response:

Topics				
1.	2.	3.	4.	5.
Interests	Sport	Future plans	Future apartment	Music

Please sloganize the four most important topics of conflicts that occurred between you.

Topics of conflicts			
1.	2.	3.	4.

Example response:

Topics of conflicts			
1.	2.	3.	4.
Different priorities	Lack of my vision for the future	Delays of the ex-partner for meetings	Feelings or lack thereof in a relationship

Spending time together

Please list five activities that you did together with your partner in spending time together. If possible, please give a brief account of them.

Activity 1, Activity 2, Activity 3, Activity 4, Activity 5) (after each "Activity" there was a field to enter the response)

Example response:

Activity 1
Dancing

Activity 2
Drinking coffee

Activity 3
Learning

Activity 4
Walking

Activity 5
Meeting with friends

Please sloganize six people or groups (e.g., my parents, my partner's parents, mutual high school/work friends, etc.) with whom you have maintained regular contact as a couple (joint meetings, conversations, trips).

People					
1.	2.	3.	4.	5.	6.

Example response:

People					
1.	2.	3.	4.	5.	6.
His friends	My friends	Friends from my school	Friends from the hair salon	My friend [comment from the first author: friend gender: female]	His friend [comment from the first author: friend gender: male]

XXXXXXXXXXXXXXXXXXXXXXXXXXXXXXXXXXXXXXXXXXXX [ ] – contensts in brakets - addition from the first author needed to understand content of participant's response]
