# Supplementary material for: The effects of narrative framing of own broken love on understanding the past and imagining the future in close relationships
Source: PLoS One. 2025 Nov 25;20(11):e0334973. doi: 10.1371/journal.pone.0334973 (PMC12646452; doi:10.1371/journal.pone.0334973)
Supplement: S2 Appendix — (PDF) [file pone.0334973.s002.pdf]

Narrative structure assessment (score 0 from the plot structuring scale)

The assessment concerns the presence of narrative structure in the descriptive response provided. The synthesis presented by Trzebiński (2014) is taken as the operational definition of a story: "The story features characters who have certain intentions and encounter obstacles to their realization. The events and actions happening in the story focus on overcoming the obstacles or influencing them, and the end of the story is success or failure in overcoming the obstacles". For a text to be considered a story, it must refer to the entire duration of the relationship (not just to individual episodes - in particular, the period of relationship deterioration and/or breakup itself) (Boyd et al., 2020; Soroko, 2010). At the same time, the presence of a narrative thread, understood as a sequence of events depicting the interaction between the intention(s) of the protagonist(s) and the complications in their realization, is necessary (Bruner, 1986; Mancuso, 1986). The plot includes cause-effect and temporal relationships between events, and can also illustrate their causal relationships (intentional; resulting from the protagonist's intentional actions directed towards the pursuit of a set goal) (Soroko, 2010).

The final decision as to whether a narrative structure is present is based on the trained coder discernment. Clues for confirming the presence of individual components can be:

INTENTION	COMPLICATION	NARRATIVE THREAD
• Participant describes her desire to be in a relationship/reasons for agreeing to enter a relationship<br>• The participant states the fact of the beginning of the relationship (in a singular or plural form – speaking from couple “we” perspective)<br>• Despite the absence of the above two elements, the participant's sequence of actions indicates that she is motivated to taking next actions related to being in a relationship<br><br>An equivalent way to construct a narrative is to base it on the intentions of the partner.<br><br>A woman's intention to be in a relationship does not have	• The participant directly states the problem/difficulty that comes in the way of her intention to be in this particular relationship<br>• If the description of the difficulty is in abstract form (it lacks detail and concreteness) then it must be so elaborate that it is intuitively easy to accept the fact of the real occurrence of difficulties in the relationship (even if it is impossible to indicate their concrete nature)	Manifestations of the presence of narrative thread - the interaction of intentions with experienced complications include:<br><br>• Present specific events in cause-and-effect and chronological order (deviations from the linear chronology of events are permissible)<br>• Provide an explanation of why the complication encountered is considered something difficult<br>• Recall of internal states* that have arisen due to the occurrence of a complication and clearly indicate that, in light of the intention, the complication is interpreted as a difficulty/challenge/obstacle<br>• Taking action by the participant to resolve the difficulties introduced by the complication<br>• Change of personal intention - declarative or visible through a change in the direction of actions taken

to be based on a desire for a close relationship, it can, for example, be instrumental (gaining support from a partner).<br><br>Particularly valuable indicators of the presence of intention (and often plot) are the evocation of internal states* arising from the impact of current events on the development/sustainment of intention.		A well-defined plot allows seeing how the characters affect the course of events and what is the role and meaning of the various elements of the story.<br><br>The reason for excluding the presence of a plot is not the abrupt termination of the relationship after complications, if the party who decided to end the relationship/responsible for it was the partner.<br><br>The sole indication that the occurrence of complications caused the breakup of the relationship is not considered a manifestation of the presence of a plot (since the interaction with the intention is missing).

In situations of significant doubt as to whether a given response has a narrative structure, impossible to judge by the criteria in the instructions, the final rationale for the decision may be to try to speculate on how the thoughts that make up the written response are constructed in the participant's mind (whether or not, when thinking about her relationship, she is creating a story that encompasses the entire relationship). [INSTRUCTIONAL ELEMENT ADDED AFTER COMPLETION OF ROUND 1 OF STUDY 2 ASSESSMENTS].

A personal narrative is not in particular:

1. reflections on the relationship

2. descriptions of episodes of relationship deterioration and/or separation

The way trained coders work:

1. Answer the question: does the answer have a narrative structure? (Is it a personal story?) (YES/NO).

***Internal states:**

Motivational internal states and processes of an affective-motivational (e.g., emotions, desires, needs, intentions, intentions, goals) or cognitive (e.g., thoughts, perceptions of situations, perspective taken, beliefs, reasons for action, values) nature.

Narrative set experimental instruction:

The story of my former relationship

We are interested in knowing your individual history of a relationship that ended in a breakup.

We want to ask you to tell it. **You will decide what you will share with us** and how you will approach telling the story of your former relationship with your partner.

We would like to know how your relationship has changed from getting to know each other through the decision to be together until the breakup.

Please write freely without considering the style or worrying about mistakes. Just as you wrote only for yourself. Most importantly, the description should be true and sincere. You may **change your partner's name and any other details** for your comfort. We would be very grateful for longer responses (i.e., more than just one or two sentences). This will allow us to gain a fuller understanding of your experiences, which is particularly important to us.

You can describe the story of your ex-relationship the way one writes book or movie stories. Please do not focus your attention on making it interesting or engaging for the potential reader. The most important thing is that it should be your story. If you do not know how to start, you can use standard formulas: "When we first met.... ", "It all started with...", or "In the beginning...".

It usually takes **approximately 15 minutes** to write such a story. However, you do not have a time limit. When you think you are ready, please start your writing.

Please write your story in the box below:

When you have finished writing, please take a moment to read the entire text. This will enable you to make final corrections and ensure that your story comprehensively and accurately reflects what happened in your ended relationship.

Then, please proceed to the next step.

****

Part not present in the coders' instructions:

The fragment highlighted in gray was present in the instructions given to the study participants, but was not included in the task instruction content received by the trained coders.

References:

Boyd, R. L., Blackburn, K. G., & Pennebaker, J. W. (2020). The narrative arc: Revealing core narrative structures through text analysis. *Science Advances*, *6*(32), 1–10. https://doi.org/10.1126/sciadv.aba2196

Bruner, J. (1986). *Actual minds, possible worlds*. Harvard University Press.

Mancuso, J. C. (1986). The acquisition and use of narrative story grammar. In T. R. Sarbin (Ed.), *Narrative psychology: The storied nature of human conduct* (pp. 91–110). Praeger Publishers/Greenwood Publishing Group.

Soroko, E. (2010). Określanie wad i zalet metod generowania autonarracji. In M. Straś-Romanowska, B. Bartosz, & M. Żurko (Eds.), *Badania narracyjne w psychologii* (pp. 101–128). Eneteia.

Trzebiński, J. (2014). Wpływ dynamiki epizodów i klarowności wątku na siłę oddziaływania historii. In D. Filar & D. Piekarczyk (Eds.), *Narracyjność języka i kultury* (pp. 35–51). Wydawnictwo Uniwersytetu Marii Curie-Skłodowskiej.
