# Supplementary material for: The effects of narrative framing of own broken love on understanding the past and imagining the future in close relationships
Source: PLoS One. 2025 Nov 25;20(11):e0334973. doi: 10.1371/journal.pone.0334973 (PMC12646452; doi:10.1371/journal.pone.0334973)
Supplement: S3 Appendix — (PDF) [file pone.0334973.s003.pdf]

The plot structuring scale / the narrative structure scale

Brief definition of scale

The degree to which the presented text has a narrative structure and content adequate to the indicated narrative theme.

Definition of scale:

The narrative presents in an integrated way the character(s), the difficult situation in which they have found themselves, and the state of consciousness accompanying them (Bruner, 1986). "The story features characters who have certain intentions and encounter obstacles to their realization. The events and actions happening in the story focus on overcoming the obstacles or influencing them, and the end of the story is success or failure in overcoming the obstacles" (Trzebiński, 2014). A highly narrative text has clearly outlined intentions of the characters and a clearly outlined complication (problem, obstacle) that stands in the way of the realization of these intentions (content from Prof. Trzebinski's narrativity scale - Młyniec, 2019; Odachowska et al., 2019). The source of complications is the occurrence of difficulties in the realization of intentions under given situational conditions, the actors' character, or the combined effect of these both (Bruner, 1986). "The role of intention and its associated complication, or the role of plot, is central to the events described. In other words, the events are about..., they are related to..., they are triggered by the fact of the appearance of the intention and the complication in the way of the realization of the intention. To put it yet another way: a good narrative contains, first of all, a description of the events and facts that make up or pertain to the plot" (Prof. Trzebinski's narrativity scale - Młyniec, 2019; Odachowska et al., 2019). A properly structured narrative presents events and episodes ordered chronologically and/or by cause and effect (including causally). "Psychological ordering, appearing in both intentionality and causality, means taking into account human intentions as determinants of action" (Soroko, 2010, p. 108) A text with a narrative structure makes visible the elements characteristic of the structure of an episode over the course of the narrative and/or individual episodes with their causal order (underlying structure: initiating event, internal reaction, attempted action, consequence) (Baerger & McAdams, 1999; Stein & Glenn, 1979). It also allows to distinguish elements considered to be components of the basic narrative structure, i.e. introduction, development, conclusion (Dimitrova & Simms, 2022; Soroko, 2010). A text with a narrative structure implements the indicated topic, presenting an elaborate description of the necessary information while avoiding side content that hinders reception (including understanding the sequential ordering of events and the main thread of the narrative).

Additional guidelines:

The final assignment of a point value is a decision of the trained coders, in which the clarity of the narrative thread should play a leading role. It is possible to recognize the value of an answer that does not meet all the criteria, if it realizes the others to a very good degree.

The lack of an introduction to the narratives (how the partners got to know each other and made the decision to enter the relationship) should result in consideration of assigning the narrative a score 1 point lower than that based on the content presented. Narratives that do not include a description of the beginning of the relationship cannot receive the maximum score on the scale (4).

The volume of a narrative cannot, by itself, be a decision criterion for placing a narrative in a particular category.

General characteristics of the response obtaining the maximum score on the plot structuring scale in terms of structure:

An answer with a high level of plot structuring depicts the process of formation and disintegration of relationships using narrative reasoning, that is, referring to showing the intentions of the characters, the complications along the way of their realization, and the consequences of the actions taken to illustrate this with specific events and actions, and the context of consciousness accompanying the characters.

Point scale:

Point scale 0-4, from 0 - lack of a narrative structure to 4 - excellent narrative structure

Score	Definition
**0**	The structure of the description does not allow the text to be considered a narrative (critical deficiencies in meeting the requirements for the presence of a narrative structure).
**1**	The description meets the basic requirements of the narrative structure, however, the narrative remains at least partially incomprehensible (e.g., vagueness of the intentions of the characters and the complications they encounter, disordered linking of events - chronological and/or cause and effect) or fragmentary (e.g., deficiencies in the presentation of the introduction, development or completion of the narrative flow with a completed link, the transmission of too little meaningful information, the transmission of off-topic content).
**2**	The description meets the requirements for the presence of a narrative structure, with an introduction, development and conclusion. The way in which events are tied together is generally good. Intention, complication and their interaction are presented, although they are not elaborated, or despite their good presentation, the description often deviates from the main theme, so that it contains a poorly outlined narrative thread. Divergence from the theme can be, for example, in the form of extended reflections that disrupt the narrative plot (sequential presentation of events).
**3**	The description meets the requirements for the presence of a narrative structure, with an introduction, development and conclusion. The way in which events are tied together is good; the chronological and/or cause-and-effect relationships between events and/or episodes are understandable. Intention, complication and their interaction are presented in a good way, creating a well-defined plot. The narrative elements described are generally on topic, and if deviating from it, do not hinder the reception of the narrative.
**4**	The description fully realizes the assumptions of the presence of a narrative structure and contains an articulated plot. Intention, complication and their interaction are presented in a broad way. The way in which events are tied together is very good - the chronological and/or cause-and-effect connections between events are clear. The narrative elements described are related to the main plot and maintain reasonable areas of volume (not too short and not too long). The process of change that the characters and/or their relationship undergo is presented while maintaining the distinct state of knowledge/awareness realistically available to the characters at the given stages of the relationship (knowledge of the ending does not determine the course of the presented story). Elements/events of the narrative have been well explained (this can be achieved, for example, through a broad representation of the area of consciousness accompanying the heroine/heroes).

Examples of assessments obtaining a particular result:

0 - lack of a narrative structure

The structure of the description does not allow the text to be considered a narrative (critical shortcomings in meeting the requirements for the presence of a narrative structure).

"It was and still is the love of my life. Unfortunately, due to reasons beyond our control, helplessness and too much time living at a distance, everything began to break down and we began to lose each other. Increasing conflicts, quarrels and misunderstandings that could not be explained by phone calls or message exchanges led to many mental breakdowns, crises, and finally to doubt, which weighed on everything. We gave each other strength, hope and a lot of positive emotions. We argued like an old married couple, many ill-considered and hurtful words were said, but when we reconciled.... and when the sun came out after the storm, we were a cure for each other. No one else and nothing else could help in worse times than we did for each other. Unfortunately, love alone is not enough. Although I know how much he loves me, I had to make this decision, because I know that it began to destroy Us all.... And He would pretend that everything is ok. I've hurt Him more than anyone else so far, but I hope that someday He will understand. Unfortunately, "all love begins with presence and comes down to presence". In Our case it was missing. All that's left is hope."

"I met my ex after a difficult relationship with a possessive and jealous person. He was the opposite - cheerful, spontaneous and sociable. The relationship ended because it turned out that he was an irresponsible, carefree person. I had to take care of all the organizational issues in the relationship and other less pleasant matters. He also had a tendency to flirt. He cheated on me. He got into debt and did not look for a better job to get out of it, preferring to do what he liked. He wasn't budging on starting a family and getting married. He said one thing and did another. He was often influenced by his colleagues. In the end, I met my current fiancé and made the decision to separate."

1 – incomplete narrative structure

The description meets the basic requirements of the narrative structure, however, the narrative remains at least partially incomprehensible (e.g., vagueness of the intentions of the characters and the complications they encounter, disordered linking of events - chronological and/or cause and effect) or fragmentary (e.g., deficiencies in the presentation of the introduction, development or completion of the narrative flow with a completed link, the transmission of too little meaningful information, the transmission of off-topic content).

"We met at the university. He came for tea and stayed. At first we had convergent plans. We went abroad together. Over time, we stopped caring about each other. In addition, his attitude about having children changed - he said that he would like to have some someday, though. Here there are no compromises, because I don't want to for sure. After many conversations, or rather monologues on my part (he couldn't and can't talk) the only way out was to break up."

"It all started when he saw me on the XXXXX, asked me out, after a fairly short time we were a couple. Everything started to break down, because we stopped meeting, even though I loved him I stated that I would somehow mobilize him by saying that it was over, I don't know why I did it myself, he pounced on it, but one day he came with a rose and I succumbed again, after days of passion we stopped meeting again, I think that love didn't die, although I don't know, in my heart it is still alive, and although I often see him at work, he treats me as an acquaintance, after all that was between us..."

2 - basic narrative structure

The description meets the requirements for the presence of a narrative structure, with an introduction, development and conclusion. The way in which events are tied together is generally good. Intention, complication and their interaction are presented, although they are not elaborated, or despite their good presentation, the description often deviates from the main theme, so that it contains a poorly outlined narrative thread. Divergence from the theme can be, for example, in the form of extended reflections that disrupt the narrative plot (sequential presentation of events).

“We knew each other for a long time, we bumped into each other in a XXXXX, that's how we started dating. After a year we moved out to a rented apartment, then we had to move out and moved to his parents' house on XXXXX, but with a shared kitchen. In the meantime, I lost my job and couldn't find a new one. His XXXXX was an alcoholic, i.e. every weekend XXXXX would get drunk, after one such weekend we quarreled and I went abroad to earn money for the apartment, the then partner had a good job and did not know XXXXX so he stayed. After a month or two of being long-distance, I began to be bothered by his constant texting and calling. I had a hunch and checked his phone records. We argued for the next two months until I finally broke up, I decided there was no point, the feeling on my part had burned out, even more so when I saw him texting with a friend after nights, at which I caught him lying, etc”.

3 - good narrative structure

The description meets the requirements for the presence of a narrative structure, with an introduction, development and conclusion. The way in which events are tied together is good; the chronological and/or cause-and-effect relationships between events and/or episodes are understandable. Intent, complication and their interaction are presented in a good way, creating a well-defined plot. The narrative elements described are generally on topic, and if deviated from, do not hinder the reception of the narrative.

"We met at XXXXX. I, at the time, was in a relationship with another man (T). We lived together, but I had been planning to break up with him for a long time. When K heard my story, he supported me in the breakup. I liked him and more than once imagined that we would be the ones together. K invited me to a movie on Valentine's Day. Despite the fact that I was in a relationship with T - I went. Nothing happened between us. In XXXXX, T finally moved out and I became free. a day later, I went with K to the cinema, then for pizza and beer. At XXXXX he found that there was no way to get home, so he went to spend the night at my place. That night we talked a lot. The next day we didn't go to work. I was his first woman in every sense. He was happy and delighted, and I felt overwhelmed by the rapid entry into a new relationship. I stayed with him because he was completely different from my ex. Besides, it gave me pleasure to have someone take such care of me. He gave me all too much, and I became increasingly angry and abusive. We went on vacation abroad together. After the vacation, our relationship improved. Although it was still not warm and loving behavior on my part. Maybe at times. At some point I decided that I could no longer treat him like this. I went to a psychologist and started therapy. I also started to get involved in the relationship and make an effort. K also began to make an effort. We started planning another vacation when I found out by accident that he had been sending nude photo requests to his girlfriends for a long time. This hurt me a lot. Although it may not have been a very serious "offense," I couldn't handle it and ended the relationship. I quietly hoped that he would try to get back to me and I knew that I would forgive him - however, he did not try. And so I was left alone."

4 – excellent narrative structure

The description fully realizes the requirements of the presence of a narrative structure and contains an well-establised plot. Intention, complication and their interaction are presented in a broad way. The way in which events are tied together is very good - the chronological and/or cause-and-effect connections between events are clear. The narrative elements described are related to the main plot and maintain reasonable areas of volume (not too short and not too long). The process of change that the characters and/or their relationship undergo is presented while maintaining the distinct state of knowledge/awareness realistically available to the characters at the given stages of the relationship (knowledge of the ending does not determine the course of the presented story). Elements/events of the narrative have been well explained (this can be achieved, for example, through a broad representation of the area of consciousness accompanying the heroine/heroes).

“We met just before the age of XXXXX, through a mutual friend. I liked him very much, in fact, at first sight. Before that I did not have a boyfriend, yes, I dated occasionally and kissed some boys, but it was not "for real". I was also his first girlfriend, and yet he was not shy, he quickly took the initiative. We talked a lot, walked, joked - we had a lot of common topics and a similar sense of humor. After about 2 months of meetings we were a couple. I'm very constant in my feelings, and once I make up my mind about someone, I get really involved. And I've always been like that, even when it came to elementary school "love", I could sigh at one friend for 3 years!!! And here, after about 2 months of the relationship, I had a "crisis" and doubts if this is definitely it. It wasn't because of another boyfriend, the behavior of the current one, etc., I guess I just had "such a whim". Fortunately, he made an effort, got involved with me, we went on walks and I quickly fell in love up to my ears. The first year of the relationship was wonderful for me: similar interests, sense of humor, he took the initiative in many things, organized trips, excursions, surprises, he was very much in love. For me, the ideal. At that time, everything about him suited me. But a year passed, we began to argue sometimes, you know, the first differences of opinion. However, the arguments always ended well and I was convinced that we were still very much in love, or at least I was. The breakthrough came after a year and 2-3 months. A former friend who had once liked him wrote to him. I was a little concerned, I argued with him about it (because I was sometimes jealous, and before that he did not speak of her in superlatives and claimed that he did not want to have contact with her), but in the end I somehow accepted it, but something changed. He started to contact her very often, once she came to our XXXXX for a week, then he met with her every day, and with me only 2 times in 7 days (usually he came every day or every 2 days) and then I got upset. We argued about it and he broke up then. And later, after 2 days he wanted to come back because he cares. I didn't want to, but my dad convinced me to "give it a chance, maybe the boy made a mistake". And so my hell began. From then on it got worse and worse between us. I had time off after high school graduation, so I spent my days going out with friends, trying not to think about our situation, he was working (because he had a year left to finish technical school, but he was making extra money during the vacations), so we saw each other a little less often. Then we went on vacation and it was terrible: a lot of arguments, indifference on his part, I often cried. After the vacation, we parted once again, because he said he didn't know if he loved me. But we came back again, because he supposedly "cared," and I was very much in love. Anyway, he kept telling me that we would fix it together, we could do it, and I believed. From our relationship became a terrible sine wave. He couldn't stand it mentally, so he became addicted to certain psychoactive substances and "excaped" into them. I often cried and walked around depressed. We broke up 2 more times, 2 times we came back. One time the breakup was with a break of several weeks to "rest". At that time he told me that everything was my fault, because I had a hard character. I decided to change and not do things that upset him and we got back together. We lasted like that for 4 months, it was once better, once worse, but without serious arguments. Only that he was less affectionate and I cried a lot because of it, I was concerned about him, where he was and what he was doing. And he dated female friends, with whom, however,

he supposedly "did nothing" because "he wouldn't know how to cheat on me." I also eliminated from my behavior all the things that annoyed him, i.e.: I didn't complain, criticize him, pay attention to him, talk about my needs, or demand anything, because it immediately annoyed him. I learned to do without tenderness, love, warmth. For a few months we lived practically as sex buddies: we met, did cool things - trips, excursions, varied time together, sex, but without much tenderness, confessions, declarations of affection. After another vacation together, things got worse, because he told me that in his opinion I had a medium figure, too small breasts and too flat a bottom (I am slim and shapely). I cried a lot, because he was the ideal for me, but he said he would motivate me to exercise to improve my figure. He motivated actually, but in the meantime he was staring at other girls on the street, and I saw it. Later he said that he was bored with sex with me and wanted to have it with someone else, because he had not yet had any partner other than me. We argued a lot, and I started thinking about breaking up. And he was offended for two days because I dared to make an argument with him! In the meantime, it turned out that he had not logged out on a certain social network on my computer, and by chance messages came to me from that former friend, with whom he was in contact all the time. In them he wrote that he wanted to have sex with her, sent her nude photos of himself and wrote that he was bored with me, but I have a cool character and he doesn't want to break up. I broke up with him right away and never came back (I didn't give the reason right away, he found out from me later), and he tried several times to come back. I suffered a lot after that breakup, but I noticed how much he terrorized me mentally, spoiled my self-esteem, put all the blame on me and took away my identity. And I let him do it. Sometimes some memories of that relationship still come back, and then I am distrustful in male-female relationships".

Example responses from the current project:

0 - lack of a narrative structure

The structure of the description does not allow the text to be considered a narrative (critical shortcomings in meeting the requirements for the presence of a narrative structure).

"I sacrificed everything for this boy because I went abroad but it taught me a lot that I can handle myself in any situation"

"We met at work and were together for a month. However, it was noticeable the lack of interest on the other side and little desire to solicit contact hence the decision to end the relationship."

1 – incomplete narrative structure

The description meets the basic requirements of the narrative structure, however, the narrative remains at least partially incomprehensible (e.g., vagueness of the intentions of the characters and the complications they encounter, disordered linking of events - chronological and/or cause and effect) or fragmentary (e.g., deficiencies in the presentation of the introduction, development or completion of the narrative flow with a completed link, the transmission of too little meaningful information, the transmission of off-topic content).

"Everything started with a conversation with a friend. She knew that I was alone and suggested introducing me to her acquaintance. We started writing on the internet and after some time we met.

At the beginning I was skeptical about this acquaintance, but with each meeting our relationship started to build and develop. He was romantic, polite, he treated me with respect. We spent a lot of time together, shared weekends. He himself encouraged a deeper relationship, we talked a lot about different topics. Everything was going in the right direction.

Suddenly I felt that something was wrong. After trying to talk with my partner, he avoided it, didn't engage in the conversation. After a moment he said goodbye and went back to his place.

He blocked me on social media without giving a reason and without explaining anything. He sent my things via a parcel locker. That's how this relationship ended."

2 - basic narrative structure

The description meets the requirements for the presence of a narrative structure, with an introduction, development and conclusion. The way in which events are tied together is generally good. Intention, complication and their interaction are presented, although they are not elaborated, or despite their good presentation, the description often deviates from the main theme, so that it contains a poorly outlined narrative thread. Divergence from the theme can be, for example, in the form of extended reflections that disrupt the narrative plot (sequential presentation of events).

"Everything started at the birthday party of one of my friends. I met her cousin there, and we quickly got along very well. We talked almost the entire party. He texted me the next day. He invited me for coffee and cake. We spent almost the whole day together.

At first, we were just friends, and we both considered each other just a pair of friends. We met up with our mutual friends, went to parties. But one day, he invited me to a wedding of his distant cousin. He said that he had no one else to ask. I agreed. We had fun until dawn. He didn't take his eyes off me.

Just before the end of the party, he asked me if I would be his girlfriend. At first, I thought it was a joke because he was slightly drunk, and I didn't take it seriously. But the next day, he asked me the same question, and I agreed.

During our relationship, we both supported each other. We were each other's support. We could count on each other. We spent a lot of time together. We never got bored with each other. But at some point in our relationship, we both clearly noticed that our priorities and interests were starting to drift apart. We still felt great together, but we saw that our feelings had faded.

So, we decided to break up and stay friends, which we are to this day. We still keep in touch and meet often (this time, however, only as friends)."

3 - good narrative structure

The description meets the requirements for the presence of a narrative structure, with an introduction, development and conclusion. The way in which events are tied together is good; the chronological and/or cause-and-effect relationships between events and/or episodes are understandable. Intent, complication and their interaction are presented in a good way, creating a well-defined plot. The narrative elements described are generally on topic, and if deviated from, do not hinder the reception of the narrative.

"We met on Tinder. At first, neither of us wanted to enter a relationship. We just wanted to be friends. However, the more we texted and met, the more we started to care about each other. We made a mutual decision that we wanted to be together.

XXXXX was a wonderful boyfriend when he was in a good mood. He could be affectionate, he could be supportive, he made an effort. But he regularly fell into what he called 'down phases.' When he was in a bad mood, he treated me like air. He was cold, he didn't want to talk about anything, he didn't see it as a problem. His answer was always, 'That's how I am.' When he was in a good mood, he would say that in a 'down phase' he wasn't himself.

I always tried to support him, to pull him out of that phase, I tried to help him because of his past. I wanted him to experience true, peaceful love. Now I see that I did it at the expense of myself. I engaged in it with my whole being, trying to bring back his good mood as quickly as possible so that he would show me how much he loved me again.

He acted like a typical toxic person. I couldn't imagine life without him. I suffered and cried every time he became cold. He broke up with me several times without informing me about it. He just decided in his head that we were no longer together but never told me. He would only say it when his good mood returned and everything was great again.

I became addicted to this emotional rollercoaster. I tried not to do anything that could put him in a bad mood. Like playing with a bomb—you never know when it will explode. It got to the point where I didn't show when I was in a bad mood or sad, just so he wouldn't feel rejected. I handled everything on my own.

His words didn't match reality at all. He said that if I had a bad mood, if I was sad, he would support me. But when it actually happened, he got offended at me and didn't try to support me in any way.

I stayed in it because I loved him deeply. The decision to break up was his. He broke up with me over Messenger, without directly saying that it was over. He claimed that he wasn't fit to be with anyone, that he needed to be alone. He said he wouldn't tell me directly that it was over because he wasn't able to say it, because I was the most important person in his life, and he wouldn't be able to live without me.

After the breakup, we still had contact, he gave me false hope that things would be fine. Then suddenly, from one day to the next, he stopped responding. About a month after the breakup, I got a Snapchat from him where he was holding hands with a new girl.

I texted him, telling him that it wasn't fair, that it was the worst thing he could have done, and that I wanted to know the real reason for the breakup. He replied that I had started to annoy him with my personality. Never before, not even in a 'down phase,' had he treated me like trash the way he did then.

He hated me. It took me a very long time to recover from this. I cried and struggled with it for about a year. What hurt the most was the fact that he moved on instantly, after claiming that he wouldn't be able to live without me, and additionally blocked me everywhere on all social media just like that.

Now I see that he actually saved me with that breakup, because I would have never left him. No one has ever broken me mentally the way he did. Despite all of this, I still wish him well because at one time, he was my whole world."

4 – excellent narrative structure

The description fully realizes the requirements of the presence of a narrative structure and contains an well-establised plot. Intention, complication and their interaction are presented in a broad way. The way in which events are tied together is very good - the chronological and/or cause-and-effect connections between events are clear. The narrative elements described are related to the main plot and maintain reasonable areas of volume (not too short and not too long). The process of change that the characters and/or their relationship undergo is presented while maintaining the distinct state of knowledge/awareness realistically available to the characters at the given stages of the relationship (knowledge of the ending does not determine the course of the presented story). Elements/events of the narrative have been well explained (this can be achieved, for example, through a broad representation of the area of consciousness accompanying the heroine/heroes).

"We met online. I was 18 at the time and he was 23. He started writing to me because earlier, on a Facebook group called "stresawka," I had vented that I was stressed about never meeting anyone (that I would never experience love in the form of a boyfriend).

We wrote to each other all day long for a month. We had shared interests (I play the cello, he plays the accordion, we both dressed in vintage styles [me – 50s, him – 30s]). After a month, we started talking on the phone. Every day it was about 2–5 hours. I kept this relationship secret from my parents for a long time because they are very strict.

After four months, we met – he came to me by train (7 hours of travel). That's when we met in person. Two months later, he came again, then a month after that, we met halfway, and from then on, we started being together. I told my parents, and they called him and agreed that since I'd be in my final year of high school after summer, he could visit me no more than every two weeks. So we met regularly every two weeks.

I had my first time (sex) with him, and also my first kiss. It was my first relationship ever. Our relationship was perfect. Unfortunately, after 10 months of bliss, things started to sour. After six months, he wanted me to move in with him and transfer to another school (distance of 477 km). I refused because my high school final exams and cello diploma were coming up, and I didn't want to change my environment.

He complained a bit, but the topic quieted down. He was getting tired of always being the one to visit me. So we planned that this time I would go to him. My parents didn't want to allow that. Mainly, they are obsessed with virginity (they didn't know I had already had sex) and didn't want "IT" to happen under any circumstances.

When my persuasive arguments didn't work, I called him and told him I did everything I could to

convince my parents. That’s when everything started to fall apart. My ex changed drastically – from a sweet, caring, and understanding boyfriend to someone else entirely.

A month before the breakup, he became extremely jealous (even though I didn’t have any male friends). He forbade me from wearing makeup (even though I only used concealer) and from talking to any boys (even classmates).

A week before I broke up with him, he kept telling me that I was not mature enough to go see him without my parents knowing. My parents had previously used physical violence on me, so I didn’t want to risk it. My ex knew I was afraid of them, but still claimed I wasn’t trying hard enough in the relationship and that I couldn’t ignore my parents.

(Every attempt to travel on my own would have ended with my mom calling the police and going across Poland to find me.) Despite knowing the full situation, he compared me to his ex, who, even though she was 15, used to travel to see him on her own (though she had a completely different family situation).

That whole week, he yelled at me and accused me of being not mature enough. Eventually, my parents agreed to let me go visit him, but on the condition that they would drive me and I wouldn’t spend the night. He didn’t like that condition.

After another conversation with my parents, I knew the situation was critical, and if I didn’t break up with him, he would dump me (I am being treated for depression, so being dumped would have affected me worse than making the decision myself).

In our final conversation, I told him thank you for those 10 months and for every minute spent together. He got angry, started calling me names, and said, “f*** off.”

An hour after the breakup, he started calling me and my parents every day and sending long messages. Even after blocking his number, he called from others. He begged me, said he would change, etc. (I didn’t believe him – once before he had an aggressive outburst, and when I told him to go to a psychologist and work through it, he said he would never want to change).

Every day I was scared he would come to my city to beg me to come back. After 3 months, the fear passed. But half a year after the breakup, when I thought he had forgotten about me, he called from another number. I picked up, didn’t recognize his voice, and heard, “Come back to me or at least let me talk to you.” I replied, “No,” and hung up. I was terrified.

It’s been 13 months. I’m no longer afraid of him, but I think I’ll never go to his city alone (he has a gun license and 5 firearms). I’m ready for a new, happy relationship with someone who will respect and empathize with me.

Admittedly, for three months after the breakup, I kept telling myself no one would love me and I’d be alone forever. But now that I’ve moved to another city for college and met lots of people, I’m almost certain that someone will love me sincerely and that I deserve that happiness.

The only thing I worry about is whether I’ll fall into another toxic relationship. But despite that, I believe my next relationship will be a happy one”.

****

Part not present in the coders' instructions:

XXXXX – section anonymized to protect the participant's privacy (trained coders read full responses)

[he] – content in brackets – addition from the project leader necessary to clarify the participant's response; may also be used for anonymization.

Trained coders used an instruction version that did not include the page number from the cited source: Soroko, 2010.

References:

Baerger, D. R., & McAdams, D. P. (1999). Life story coherence and its relation to psychological well-being. *Narrative Inquiry*, *9*(1), 69–96. https://doi.org/10.1075/ni.9.1.05bae

Bruner, J. (1986). *Actual minds, possible worlds*. Harvard University Press.

Dimitrova, J., & Simms, L. J. (2022). Construct validation of narrative coherence: Exploring links with personality functioning and psychopathology. *Personality Disorders: Theory, Research, and Treatment*, *13*(5), 482–493. https://doi.org/10.1037/per0000508

Młyniec, A. (2019). *Narracyjna Kontrola Zachowania w obszarze studiowania i bliskich związków [Narrative action control in studies and romantic relationships]*. Doctoral dissertation, SWPS University of Social Sciences and Humanities.

Odachowska, E., Trzebiński, J., & Prusik, M. (2019). The Impact of Self-Narrative Framing of a Close Person's Sudden Death on Coping With the Meaning in Life. *Journal of Loss and Trauma*, *24*(4), 293–321. https://doi.org/10.1080/15325024.2019.1565145

Soroko, E. (2010). Określanie wad i zalet metod generowania autonarracji. In M. Straś-Romanowska, B. Bartosz, & M. Żurko (Eds.), *Badania narracyjne w psychologii* (pp. 101–128). Eneteia.

Stein, N., & Glenn, C. (1979). An analysis of story comprehension in elementary school children. In R. Freedle (Ed.), *New directions in discourse processing (Vol. 2)* (pp. 53–120). Ablex.

Trzebiński, J. (2014). Wpływ dynamiki epizodów i klarowności wątku na siłę oddziaływania historii. In D. Filar & D. Piekarczyk (Eds.), *Narracyjność języka i kultury* (pp. 35–51). Wydawnictwo Uniwersytetu Marii Curie-Skłodowskiej.
