# Supplementary material for: The effects of narrative framing of own broken love on understanding the past and imagining the future in close relationships
Source: PLoS One. 2025 Nov 25;20(11):e0334973. doi: 10.1371/journal.pone.0334973 (PMC12646452; doi:10.1371/journal.pone.0334973)
Supplement: S4 Appendix — (PDF) [file pone.0334973.s004.pdf]

Coherence of breakup reasons description

<table><tr><td>
Study question:
“Why did your relationship end?
Please answer freely, as if you were writing for yourself. Without caring about style or worrying about mistakes. The most important thing is that the thoughts conveyed are genuine and sincere. We ask that you take as much time as you deem necessary to fully present your thoughts”.
</td></tr></table>

The task of the trained coder is to evaluate the participant's answer in terms of the coherence of the argumentation contained in it. The individual components in a coherent text are interrelated, do not contradict each other, and fit into the context of the utterance and its topic (simplified definition after Reinhart 1980; after Wang and Guo, 2014). Coherence refers to both the content structure of an utterance (the connections between elements present in it) and the ease with which the intended meaning can be read by the recipient (Wang and Guo, 2014).

In the evaluation, we assume that the content given by the participant as factors leading to the breakup is true.

The scale used is 5 points.

The description is characterized by .... coherence of argumentation.

1 - absent
2 - very low

3 - sufficient

4 - good

5 - very good

1 - absent
The description does not contain valuable argumentative units. It is abbreviated and consists of, for example:

- generalities ("it wasn't it", "love died")
- pseudo-reasons (elements that occur in every relationship, e.g., "we were different from each other"), arguments of very poor value (e.g., referring to romantic love scripts "I was not perfect for my partner")
- declaring lack of knowledge as to the reasons for separation
- content indicating considerable uncertainty about the reasons for the separation, expressing conjectures whose purpose seems to be not to consider alternatives, but to give the first better possible reason
- the slogan assignment of all responsibility to the partner

2 - very low

The description is based on a synthetic listing of the cause or causes (e.g., in the form of categories grouping the experiences and phenomena responsible for the separation). The causes that occur **<u>are</u>**

not related to each other. Stating the same argument in other words is not considered an extension of the description (e.g., "I think because of the mismatch, we were too different.").

3 - sufficient

The description includes a representation of the relationships between elements and/or their detailing.

The quality of the inference is not good, due to: poor logical quality of the stated connections between elements, lack of logical structure of the entire statement (e.g., resulting from devoting a significant amount of text to content not directly related to the inference - such as descriptions of personal experiences, reflections that do not support the value of the argument).

4 - good

The description is characterized by a good representation of the relationships between the elements or a good detailing of them (stating what qualities the element has to show why it should be considered significant for the separation).

5 - very good

The description is characterized by a very good representation of the relationships between the elements (logical structure) and their high level of detail (stating why the element(s) should be considered significant for separation).

In addition, descriptions unsuitable for evaluation (careless, unintelligible, containing a refusal to answer) are distinguished.

Coherence of argumentation	Sample answers from the level
1 - absent	"We were not a good match."<br><br>"To the end, I myself do not understand why it happened. We didn't fully explain it to each other. I think he wasn't ready for a relationship."<br><br>"Its end was partner's responsibility, he prefers bachelor's life 🙂 "
2 - very low	"Betrayal"<br><br>"Different priorities, visions of the future, ways of spending leisure time, getting used to each other, lack of love (?)."<br><br>"Because of the difference in characters and lack of time for each other."<br><br>"I think because of the mismatch, there was too much difference between us."<br><br>"Divergence about the future of the relationship on issues of shared housing. In addition, different levels of emotional commitment."
3 - sufficient	"Our relationship fell apart primarily due to the fault of the partner [he] totally did not try for us to compete for love it was weak [in meaning of: disappointing] on his part."

	"I think it was meant to be that way. It's just that his approach was not mature and he didn't care about me. And it was a good thing after a while I found that I was fine on my own and we were not a good match."<br><br>"I had had enough. I felt from the beginning that this was not it, but I thought maybe it was normal and it was supposed to be like this. At some point I just already knew that I didn't want to be with him because we were too different and had very little in common and a different approach to life."<br><br>"I ended this pseudo-relationship because I realized how devastating it was to my psyche. it took a third party to help me see this, but I am perfectly happy that our paths parted. my ex wanted to have full control over what I do and who I meet. The most he would do is to keep me locked in one room. fortunately, this is behind me and now I am finally happy."<br><br>"The first week at the university was something scary for me. I asked for time for me because I wanted to recognize [in meaning of understand] myself in this new experience.<br>However, he said that since I couldn't meet with him during the week, it meant that I no longer had time for him, and he didn't want to continue.<br>Authentic story. I have evidence and screenshots from XXXXX .... :("<br><br>"In fact, the end of our relationship lasted two weeks, it was a very difficult two weeks during which I took tranquilizers, starved myself and my partner remained indifferent to my feelings, did not speak, did not write did not call. After those two weeks he said via text message that he wanted to end the relationship because he wanted to be free and wanted to be a bachelor (three weeks later he was already dating another)."
4 - good	"I couldn't cope with his gambling anymore. I couldn't help him, it finished me mentally."<br><br>"My partner cheated on me with his previous girlfriend. I had already forgiven him for cheating once, the second time I couldn't."
5 - very good	"The relationship ended because I didn't want to experience the same thing as in an even previous relationship - physical betrayal. I felt that my partner betrayed me emotionally, and despite

	explaining to him what constitutes betrayal to me and establishing boundaries in the relationship, he did not respect it. He dated other women behind my back, lied and avoided contact. I respect myself enough that I won't allow myself to be treated that way and don't want to live with someone who doesn't respect me."<br><br>"He made the decision to study in another city, I could not go with him at the time. He doesn't believe in long-distance relationships, and due to the fact that I could only be in one at the time, we parted ways."<br><br>"He didn't feel anything for me and that I can understand, sometimes that happens in life. I just have a problem understanding why he behaved the way he did and why he didn't tell me about it right away (he hid behind friendship, but I guess friends are honest with each other, right?)”.

****

Part not present in the coders’ instructions:

[ ] – content in brackets – addition from the first author necessary to clarify the participant’s response.

XXXXX – section anonymized to protect the participant’s privacy.

References:

Wang, Y., & Guo, M. (2014). A short analysis of discourse coherence. *Journal of Language Teaching and Research, 5*(2), 460–465. https://doi.org/10.4304/jltr.5.2.460-465
