# Supplementary material for: The effects of narrative framing of own broken love on understanding the past and imagining the future in close relationships
Source: PLoS One. 2025 Nov 25;20(11):e0334973. doi: 10.1371/journal.pone.0334973 (PMC12646452; doi:10.1371/journal.pone.0334973)
Supplement: S5 Appendix — (PDF) [file pone.0334973.s005.pdf]

The theme of thoughts on the future in close relationships

Study question:
**"Thoughts on the future in close relationships**
Please share your thoughts over the past week about your future in close relationships".

The task of the trained coder is to classify the participant's answers into one of the distinguished categories according to the definitions described in the table. It is recommended to assign answers to only one category and to use a mixed category only if necessary.

**Category group**	**Category**	**Definition of**	**Examples**
Interest to think about the future:<br>The description presents ideas of a possible future or plans for the future.	Focus on personal intentions	Description focused on personal intentions. Manifestations of focus on personal intentions include:<br>• declaring (explicitly or between the lines): discovered values, personal standards, hopes held, aspirations in future relationships, motivations for action<br>• Presenting plans for building future relationships (including solving possible problems)<br>• Mentally simulating specific actions to enter a relationship (e.g., ways to get to know a prospective partner).	"I already know what I want. What qualities my future partner must and must not have."<br>"I wondered what the next relationship could look like, what I would pay attention to in a partner, what I would change in my behavior. I think I have learned various lessons and lessons from my previous relationship, and I would like to use this in my next one."<br>"I think in the future in a relationship I will be more careful before trusting someone; it's better to get to know someone first than to regret it later."<br>"I would like to create the kind of relationship that is based on trust."
	Neutral	The description presents thoughts on the future having the form of general considerations about it.<br>These thoughts differ from those focused on personal intentions in that:<br>• do not directly describe the desired states	"Will it still be possible to form a close relationship?"<br>"Thoughts about what will be, what my life will be like someday, whether I will find a partner someday, that kind of thinking about what

		- do not contain premises that allow the assumption that the desired states/actions are specified in the mind of the participant - are not operational in nature (they are not plans or mental simulations of specific actions aimed directly at creating a relationship). <br>Instead, they may involve trying to determine personal values and ways of doing things (e.g., undirected consideration of what qualities a partner would be worth seeking).<br><br>In contrast, problem-focused thoughts are distinguished from problem-focused thoughts by the lack of thematic dominance of the problem. The theme of the problem/challenge may be present, but it does not entail fear and a pessimistic vision of the future.	it will be like with me in the future."<br><br>"Because of the planned wedding in my family, I began to think more intensely about a possible future relationship, what it could look like and with whom I would like to create it."<br><br>"Relationships are often compromises, it's just whether I can afford them."
	Focus on the problem	The description focuses on the anticipated personal problem/challenge. The problem has a dominant role. This is evident, among other things, in thoughts depicting fears (e.g., about the chances of forming a relationship) and/or containing a pessimistic vision of the future e.g., filled with struggling, anticipating failure to achieve one's goals, dominated by personal weaknesses and fears).	"I'm afraid I'll never fall in love again."<br><br>"I don't know what my future relationships will look like. It's very possible that there won't be any, because I'll be bemused to meet someone and get involved."<br><br>"I'll probably be OVERLY jealous and constantly worry about whether I'm enough for my partner."
Reluctance to think about the future	No thoughts on close relationships	The description includes a declaration of no or few thoughts on close relationships.	"I hadn't thought about it."<br><br>"I haven't."<br><br>"There weren't that many."

	Lack of interest in romantic relationships	The description contains a firm statement of lack of interest in entering into romantic relationships in the imagined future, or does not place this as an important goal.	"I definitely won't get involved with someone soon. I don't want to waste ANY [more] time."<br><br>"I'm not going to get tied up."<br><br>"For now, I am happy to be alone. Now I see what is happening these days , I find that I prefer to be alone."
	Anchoring to the past	The description contains only references to the past (e.g., past self, partner, relationship).	"I won't trust anyone but him."
Imagined helplessness in forming close relationships	Helplessness	The description includes declaring one's perceived inability to form another relationship evident through evoked evaluations (of oneself, relationships, other elements). The description does not include elements showing forward-looking thoughts.	"I'm not fit for them".<br><br>"I'm not suitable for relationships.<br>I can't imagine myself as a wife.<br>I don't want commitments.<br>No one can really fully understand me."
Other	Mixed	The description falls under more than one category.	NOTE: categories in the area of interest in thinking about the future have a leading position over categories in the area of reluctance to think about the future.<br>If they are found together, we classify the answer as indicating interest in future thinking.
	Other	The description does not fit into any of the categories. It is understandable and contains valuable content for further analysis.	e.g., the description includes a list of emotions experienced by the participant.
	Not suitable for evaluation	Description indicating carelessness or reluctance to communicate the answer.	e.g., the description is incomprehensible, contains a declaration of outright unwillingness to answer the question.

****

Part not present in the coders’ instructions:

[ ] – content in brackets – addition from the first author necessary to clarify the participant’s response.

XXXXX – section anonymized to protect the participant’s privacy.
